# Three New Xanthones from *Hypericum scabrum* and Their Quorum Sensing Inhibitory Activities against *Chromobacterium violaceum*

**DOI:** 10.3390/molecules27175519

**Published:** 2022-08-27

**Authors:** Li-Ping Teng, Hong Zeng, Cai-Yan Yang, He-Bin Wang, Zhong-Bo Zhou

**Affiliations:** 1 Research Center for the Prevention and Treatment of Drug Resistant Microbial Infecting, Youjiang Medical University for Nationalities, Baise 533000, China; 2 School of Pharmacy, Youjiang Medical University for Nationalities, 98 Chengxiang Road, Baise 533000, China; 3 College of Chemical Engineering and Technology, Tianshui Normal University, Tianshui 741000, China

**Keywords:** xanthonolignoids, xanthones, *Hypericum scabrum*, quorum sensing inhibitory activities

## Abstract

Quorum sensing (QS) plays an important role in the production of virulence factors and pathogenicity in pathogenic bacteria and is, therefore, a hopeful target to fight against bacterial infections. During our search for natural QS inhibitors, two new xanthonolignoids (**1** and **2**), each existing as a racemic mixture, one new simple oxygenated xanthone (**7**), and eight known analogs (**3**–**6**, **8**–**11**) were isolated from *Hypericum scabrum* Linn. Chiral separation of **1** yielded a pair of enantiomers **1a** and **1b**. The structures of these compounds were elucidated by spectroscopic analysis and ECD (electrostatic circular dichroism) calculations. All isolates were evaluated for their QS inhibitory activity against *Chromobacterium violaceum*. Both **9** and **10** exhibited the most potent QS inhibitory effects with minimum inhibitory concentration (MIC) and minimum bactericidal concentration (MBC) values of 31.25 and 62.5 μM, respectively. Crystal violet staining was used to further evaluate the biofilm inhibition potential of compounds **7**, **9** and **10**, and the formation of biofilms increased with decreasing drug concentration in a classic dose-dependent manner. The results of a cytotoxicity assay revealed that compounds **7**, **9** and **10** exhibited no cytotoxic activity on PC-12 cells at the tested concentration.

## 1. Introduction

Microbial infection is still a major public health concern worldwide. Interfering with quorum sensing (QS) systems that regulate the expression of many genes associated with the virulence of pathogens was a promising alternative strategy for controlling bacterial infection, especially for those caused by multi-drug resistant strains [1,2]. *Chromobacterium violaceum* ATCC12472 is one of the most commonly used bacterial species for QS research. Quorum sensing inhibitors (QSIs) are different from conventional antibiotics in that they focus on reducing the virulence of bacteria without killing bacteria or inhibiting bacterial growth. Recently, there has been increasing evidence that phytochemicals can be an important source of QSIs [3,4].

**Figure 1:**
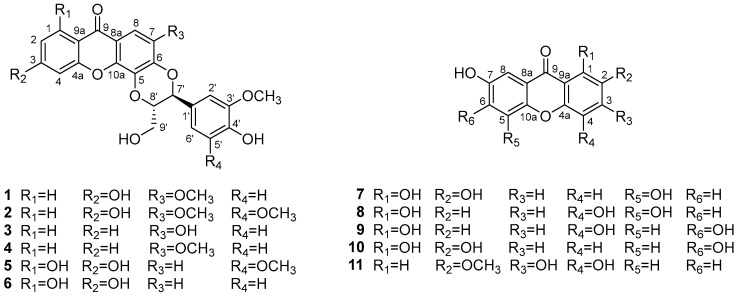
Structures of compounds **1**–**11**.

Xanthones are the main secondary metabolites of plants belonging to the genus *Hypericum*, which is widely distributed in temperate zones of the world and many have been used as folk medicines. Xanthonolignoids are a relatively rare class of natural compounds, most of which have a phenylpropane moiety attached to the xanthone skeleton through a dioxane ring [5,6]. *Hypericum scabrum* is a perennial herb growing mainly in arid rocky hillsides or gravel-sloped lands of Altai in Xinjiang, China and Central Asia, where it is commonly used to treat a variety of disorders of the heart, liver, gallbladder, intestines and bladder [7,8]. In our ongoing research for natural QSIs, eleven xanthones, including two new xanthonolignoids and one new simple oxygenated xanthone (Figure 1), were obtained from the whole plants of *H. scabrum*. Herein, we describe the isolation, structure elucidation, and QS inhibitory activities of these compounds against *C. violaceum*.

## 2. Material and Methods

### 2.1. General Experimental Procedure

UV spectra were measured on a Shimadzu UV-1800 spectrophotometer. A Thermo Scientific Nicolet iN10 Microscope was used to obtain IR spectra. ECD spectra were measured on a JASCO J1500 CD spectrometer. NMR spectra were acquired in DMSO-*d*_6_ at 303 K on a Bruker AVANCE III-500 (^1^H NMR, 500 MHz; ^13^C NMR, 125 MHz) with TMS as the internal standard. HRESIMS data were acquired on a Thermo Q-Exactive orbitrap mass spectrometer. Preparative HPLC was performed on a Shimadzu HPLC system consisting of an LC-6AD pump and a Shimadzu SPD-20A detector, coupled with a Shim-park RP-C18 column (5 μm, 200 × 20 mm i.d., Shimadzu, Kyoto, Japan). A Lux Cellulose-2 column (5 μm, 250 × 4.6 mm i.d., Phenomenex, Torrance, CA, USA) was used for chiral separation. Silica gel (200–400 mesh, Qingdao Marine Chemical Co., Ltd.), ODS (40–63 μm, Fuji, Minato, Japan) and Sephadex LH-20 (Pharmacia, Stockholm, Sweden) were used for open column chromatography (CC).

### 2.2. Plant Material

The whole plants of *Hypericum scabrum* Linn. were collected at Yumin, Xinjiang Uygur Autonomous Region, People’s Republic of China, in May 2015 and identified by Dr. Ji-Ye Liang, College of Plant Sciences, Tarim University. A voucher specimen (No. YMUN-201501) was deposited in Research Center for the Prevention and Treatment of Drug Resistant Microbial Infecting, Youjiang Medical University for Nationalities.

### 2.3. Extraction and Isolation

The air-dried and powdered whole plants of *H. scabrum* (7.9 kg) were extracted with 95% aqueous EtOH (3 × 20 L) under reflux. Removal of the solvent under reduced pressure gave the crude extract (734 g), which was then suspended in H_2_O (2 L) and successively partitioned with petroleum ether (3 × 2 L) and ethyl acetate (3 × 2 L). The ethyl acetate extract (179.8 g) was subjected to silica gel CC elution with a gradient of petroleum ether−ethyl acetate (1:0 to 0:1) to give five fractions (A–E). Fraction D (23.8 g) was separated by silica gel CC (petroleum ether−acetone, 5:1, 3:1 and 1:1; ethyl acetate; methanol) to give eight fractions (D1–D8). Fraction D5 (8.0 g) was further separated by LH-20 CC (CHCl_3_−MeOH, 1:1) to afford three fractions (D5a–D5c). Fraction D5c (1.1 g) was separated by MPLC using ODS CC (35% MeOH in H_2_O) and further purified by preparative HPLC (40% MeOH in H_2_O containing 0.1% formic acid, 10 mL/min) to yield **9** (20.4 mg), **10** (6.5 mg) and **11** (5.5 mg). Fraction E (36.8 g) was subjected to silica gel CC eluting with a gradient of methylene dichloride−methanol (20:1 to 0:1) to yield seven fractions (E1–E7). Fraction E2 (3.2 g) was purified by MPLC using ODS CC (MeOH–H_2_O, 50:50 to 100:0) to yield three fractions (E2a–E2c). Fraction E2b (439 mg) was purified by HPLC (50% MeOH in H_2_O containing 0.1% formic acid, 10 mL/min) to afford **4** (8.6 mg).

Fraction E3 (0.9 g) was separated by ODS MPLC (MeOH–H_2_O, 50:50 to 100:0) to yield three fractions (E3a−E3c). Fraction E3a (260 mg) was separated by HPLC (40% MeOH in H_2_O containing 0.1% formic acid, 10 mL/min) to afford **7** (6.2 mg) and **8** (2.7 mg). Fraction E3b (619 mg) was separated by HPLC (55% MeOH in H_2_O containing 0.1% formic acid, 10 mL/min) to afford six fractions (E3bI−E3bVI). Fraction E3bV (17.6 mg) was purified by preparative HPLC (30% CH_3_CN in H_2_O containing 0.1% formic acid, 10 mL/min) to give **1** (4.2 mg). Compound **1a** (0.7 mg) and **1b** (0.6 mg) were obtained by using a Lux Cellulose-2 column (88% CH_3_CN in H_2_O containing 0.1% CH_3_COOH, 2 mL/min). Fraction E3bIV (20.8 mg) was purified by preparative HPLC (30% CH_3_CN in H_2_O containing 0.1% formic acid, 10 mL/min) to yield **2** (1.4 mg) and **3** (1.4 mg). Finally, compounds **5** (2.3 mg) and **6** (2.1 mg) were obtained from Fraction E3bVI (412 mg) by HPLC (30% CH_3_CN in H_2_O).

6-Hydroxy-kielcorin (**1**): white amorphous powder; ^13^C and ^1^H NMR data, see Table 1; HRESIMS *m*/*z* 451.1043 [M − H]^−^ (calcd for C_24_H_19_O_9_, 451.1029).

(+)-6-Hydroxy-kielcorin (**1a**): UV (MeOH) λ_max_ (log *ε*) 244 (4.28), 321 (3.97) nm; [*α*${]}_{D}^{20}$+29.4 (*c* 0.1, CH_3_OH); ECD (*c* = 3.0 × 10^−4^, MeOH) λ_max_ nm (Δ*ε*) 379 (+0.28), 342 (+5.5), 302 (−0.91), 293 (+0.66), 289 (+0.55), 280 (+3.6), 269 (+0.44), 253 (+7.5), 235 (−7.3), 219 (+17.3), 202 (−64.4); IR *ν*_max_ 3386, 2934, 1613, 1572, 1521, 1477, 1439, 1399, 1283, 1259, 1244, 1179, 1136, 1115, 1060, 891, 846, 774 cm^−1^; HRESIMS *m*/*z* 475.1003 [M + Na]^+^ (calcd for C_24_H_20_O_9_Na, 475.1000).

(−)-6-Hydroxy-kielcorin (**1b**): UV (MeOH) λ_max_ (log *ε*) 244 (4.14), 321 (3.82) nm; [*α*${]}_{D}^{20}$−31.2 (*c* 0.1, CH_3_OH); ECD (*c* = 3.0 × 10^−4^, MeOH) λ_max_ nm (Δε) 394 (−0.12), 345 (−6.2), 304 (+0.54), 280 (−4.1), 268 (−1.1), 252 (−7.0), 236 (+5.2), 219 (−16.4), 200 (+49.6); IR *ν*_max_ 3395, 2925, 1612, 1521, 1476, 1439, 1363, 1284, 1260, 1245, 1180, 1137, 1115, 1060, 1034, 956, 891, 846, 774 cm^−1^; HRESIMS *m*/*z* 475.1001 [M + Na]^+^ (calcd for C_24_H_20_O_9_Na, 475.1000).

6-Hydroxy-3′-methoxy kielcorin (**2**): white amorphous powder; UV (MeOH) λ_max_ (log *ε*) 243 (4.11), 320 (3.78) nm; IR *ν*_max_ 3356, 2919, 1597, 1427, 1263, 1160, 1112, 1060, 877 cm^−1^; ^13^C and ^1^H NMR data, see Table 1; HRESIMS *m*/*z* 505.1105 [M + Na]^+^ (calcd for C_25_H_22_O_10_Na, 505.1105).

1,2,5,7-Tetrahydroxyxanthone (**7**): pale yellow amorphous powder; UV (MeOH) λ_max_ (log *ε*) 236 (4.30), 293 (3.88), 332 (3.88), 383 (3.93) nm; IR *ν*_max_ 3377, 1651, 1612, 1574, 1477, 1288, 1169, 1138, 1051, 801 cm^−1^; ^13^C and ^1^H NMR data, see Table 1; HRESIMS *m*/*z* 259.0248 [M − H]^–^ (calcd for C_13_H_7_O_6_, 259.0237).

1,4,5,7-Tetrahydroxyxanthone (**8**): pale yellow amorphous powder; UV (MeOH) λ_max_ (log *ε*) 228 (4.07), 255 (4.00), 283 (3.90), 390 (3.71) nm; IR *ν*_max_ 3401, 2927, 1612, 1582, 1479, 1281, 1237, 1190, 1154, 1059, 1000, 818 cm^−1^; ^13^C and ^1^H NMR data, see Table 1; HRESIMS *m*/*z* 259.0248 [M − H]^–^ (calcd for C_13_H_7_O_6_, 259.0237).

### 2.4. Anti-QS Assay

*C. violaceum* ATCC12472 provided by Ocean University of China was used as the target bacterium. The antibacterial activities of isolated compounds (**1**–**11**) were screened according to Clinical and Laboratory Standards Institute (CLSI) guidelines. For the QS assay, 100 μL of different concentrations of each compound (0–1 mmol/L) and *C. violaceum* were incubated into a 96-well plate at 30 °C for 24 h without shaking,. Then, the OD_590_ values were measured using a microplate reader (Thermo Fisher), and the minimum inhibitory concentration (MIC) and minimum bactericidal concentration (MBC) values were determined.

To quantify violacein production in *C. violaceum* after treatment with tested compounds, microorganisms in each well were collected and centrifuged for 10 min at 13,000 rpm. After removing the supernatant, an equal volume DMSO was added to precipitates to dissolve the violacein. The solution was then vortexed at maximum velocity for 1 min; the extracted violacein was acquired after another centrifugation (13,000 rpm for 10 min). The absorbance of violacein solution was recorded with a spectrophotometer at 585 nm. The inhibition percentage was calculated based on the formula reported in literature [9].

Inhibition rate (%) = (OD_0_ − OD_1_)/OD_0_ × 100%.

OD_0_ means OD_585_ of control group. OD_1_ represents OD_585_ of treated groups. Each experiment was performed in triplicate by three independent experiments.

To further study their antibacterial activity, inhibition of biofilm formation experiments of compounds **7**, **9** and **10** were conducted using a crystal violet assay. Briefly, tested compounds and bacterial suspension were mixed and added into each well of the 96-well microplate. After incubation for 16 h at 37 °C, the medium was removed and washed with sterile water three times. The plates were dried at 65 °C, and 200 µL of 1% (m/v) aqueous solution of crystal violet (CV) was added to stain for 20 min, then thoroughly washed with PBS three times. Subsequently, 100 µL of 95% ethanol was added to each well to dissolve CV at 37 °C for 30 min. The absorbance at OD 470 nm was then measured to determine the biofilm biomass on a microplate reader [2].

### 2.5. Cell Vitality Assay

PC-12 cells were cultured in DMEM containing 100 mg/mL streptomycin, 100 units/mL penicillin, and 10% (v/v) FBS at 37 °C and 5% CO_2_. PC-12 cell viability was evaluated with 3-(4,5-dimethylthiazole-2-yl)-2,5-diphenyltetrazolium bromide (MTT). Cells were grown in 96-well plates at an initial density of 1 × 10^4^ cells/well for 24 h. Then, the cells were subjected to different concentrations of compounds 7, 9 and 10 (0, 0.97, 1.95, 3.91, and 15.63 µM) for 24 h at 37 °C. Subsequently, the cells were treated with MTT (5 mg/mL in PBS) for 4 h, and a microplate photometer was used for evaluating absorbance at 550 nm.

### 2.6. ECD Calculations

Conformational analyses were carried out using the MMFF94S force field with an energy cutoff of 2.5 kcal/mol. Subsequently, the low-energy conformers were re-optimized using DFT at the B3LYP/6-311+G(d,p) level in MeOH using the polarizable conductor calculation model (CPCM). The energies, oscillator strengths, and rotational strengths (velocity) of the first 30 electronic excitations were calculated using the TDDFT methodology at the B3LYP/6-311+G(d,p) level in MeOH. The ECD spectra were simulated by the overlapping Gaussian function (half the bandwidth at 1/e peak height, sigma = 0.20 for all) [10]. To get the final spectra, the simulated spectra of the conformers were averaged according to the Boltzmann distribution theory and their relative Gibbs free energy (∆G).

## 3. Results and Discussion

**Figure 2:**
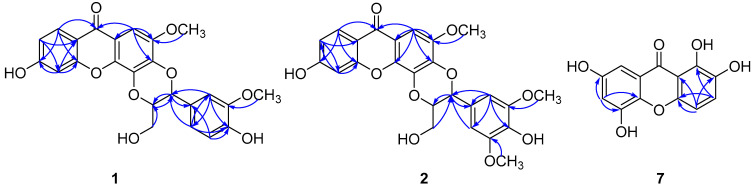
Selected HMBC (arrow) correlations of compounds **1, 2** and **7**.

Compound **1** was isolated as an amorphous white powder. Its molecular formula was assigned as C_24_H_20_O_9_ on the basis of the HRESIMS (Figure S3) ion peak [M − H]^–^ at *m*/*z* 451.1043 (calcd for C_24_H_19_O_9_, 451.1029). The UV spectrum showed absorption maxima at 244 and 321 nm. The ^1^H NMR spectrum exhibited signals of several aromatic protons, including a singlet at *δ*_H_ 7.16 and those ascribed to two ABX systems [*δ* 8.00 (1H, d, *J* = 8.5 Hz), 7.05 (1H, d, *J =* 1.5 Hz), 6.90 (1H, dd, *J* = 8.0, 1.5 Hz); 6.86 (1H, dd, *J =* 8.5, 1.5 Hz) and 6.82 (2H, overlapped)]. In addition, signals of two oxygenated methines [*δ* 5.05, (1H, d, *J =* 7.5 Hz) and 4.37 (1H, m)], two methoxy groups [*δ* 3.84 (3H, s) and 3.78 (3H, s)], and a hydroxymethyl group [*δ* 3.70, (1H, dd, *J* = 12.5, 1.5 Hz) and 3.42 (1H, dd, *J* = 12.5, 4.0 Hz)] were observed. These observations suggested that **1** was a xanthonolignoid. Overall, the 1D NMR data of **1** were highly similar to those of known compound kielcorin (**4**) [11,12]. The only difference was that **1** possesses an additional hydroxyl group, which was deduced to be at C-3 based on the HMBC correlations as shown in Figure 2. The HMBC spectrum also showed correlations from H-2′ (*δ*_H_ 7.05) to C-7′, from H-6′ (*δ*_H_ 6.90) to C-7′ and from H-7′ (*δ*_H_ 5.05) to C-1′, confirming that the trisubstituted benzene ring attached at C-7′. Furthermore, HMBC cross-peaks from H-7′ (*δ*_H_ 5.05) to C-8′, from H-8′ (*δ*_H_ 4.37) to C-7′ and from H-9′ (*δ*_H_ 3.70 and 3.42) to C-8′, suggesting that a hydroxymethyl unit linked at C-8′.

**Figure 3:**
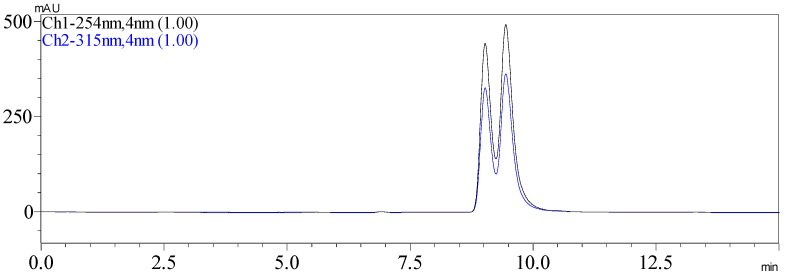
HPLC chromatogram of (±)-6-hydroxy-kielcorin (**1**) on a chiral column.

**Figure 4:**
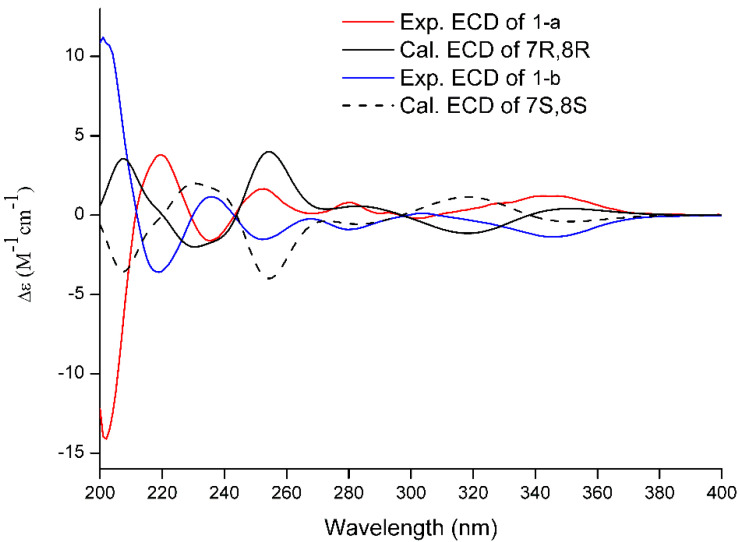
Experimental ECD spectra (200–400 nm) of **1** in MeOH and the calculated ECD spectra of the model molecules of **1** at the B3LYP/6-311+G(d, p) level.

Since compound **1** showed no Cotton effect in the ECD spectrum, it was very likely that it was a racemic mixture, which was consistent with the optical rotation value of +0.7. Subsequent chiral resolution of **1** yielded a pair of enantiomers **1a** and **1b** in an approximate ratio of 1:1 (Figure 3), which exhibited mirror image-like ECD curves (Figure 4). The absolute configurations of **1a** and **1b** were defined as (7R, 8R) and (7S, 8S), respectively, by means of ECD calculations.

Compound **2** had a molecular formula of C_25_H_22_O_10_ as deduced from its HRESIMS (Figure S8) data (*m*/*z* 505.1105 [M + Na]^+^, calcd for C_25_H_22_O_10_Na, 505.1105). The ^1^H NMR data of **2** revealed typical resonances of a xanthonolignoid, including those of a 1,3,4-trisubstituted aromatic ring [*δ* 7.97 (1H, d, *J* = 8.5 Hz), 6.84 (1H, dd, *J* = 8.5, 1.5 Hz) and 6.82 (1H, overlapped)], a penta-substituted aromatic ring [*δ* 7.16 (1H, s)], a tetra-substituted aromatic ring [*δ* 6.77 (2H, s)], two oxygenated methines [*δ* 5.04, (1H, d, *J =* 7.5 Hz) and 4.42 (1H, m)], three methoxyl groups [*δ* 3.85 (3H, s) and 3.78 (6H, s)] and a hydroxymethyl group [*δ* 3.72, (1H, dd, *J* = 12.5, 1.5 Hz) and 3.45 (1H, dd, *J* = 12.5, 4.0 Hz)]. A comparison of the NMR data of **2** with those of **1** revealed that **2** was the 5′-methoxy derivative of **1**, which was supported by the HMBC data (Figure 2). Accordingly, the structure of **2** was elucidated as 6-hydroxy-5′-methoxy kielcorin. Based on the optical rotation value of +1.1, compound **2** was also considered as a racemic mixture. However, due to the limited amount available, chiral resolution of this compound was not performed.

The molecular formula of compound **7**, C_13_H_8_O_6_, was deduced on the basis of the HRESIMS (Figure S13) ion peak [M − H]^–^ at *m*/*z* 259.0248 (calcd for C_13_H_7_O_6_, 259.0237). The UV spectrum showed absorptions at 236, 293, 332 and 383 nm, indicating that **7** was a xanthone [12,13,14]. The 1D NMR data of **7** were comparable to those of 1,2,6,7-tetrahydroxyxanthone (**9**) [15]. The only difference was that the hydroxyl group at C-6 in **9** was placed at C-5 in **7**, which was confirmed by analysis of HMBC data (Figure 2). Therefore, the structure of **7** was concluded to be 1,2,5,7-tetrahydroxyxanthone.

In addition to **4** and **9**, the other known compounds were identified as 2-demethylkielcorin (**3**), cadensin G (**5**), 5′-demthoxycadensin G (**6**) [16], 1,4,5,7-tetrahydroxyxanthone (**8**), 1,4,6,7-tetrahydroxyxanthone (**10**) [15] and 3,4-dihydroxy-2-methoxyxanthone (**11**) [17] by comparison of their spectroscopic data with those reported. Notably, the UV, IR and NMR data of **8** are reported for the first time in this work.

The genus *Hypericum* is rich in xanthones that can be classified into xanthonolignoids, prenylated and simple (hydroxy and/or methoxy) xanthones [16], which were reported to possess various bioactivities [18] such as antibacterial activity [19,20,21], cytotoxicity, etc. Since *H. scabrum* has been proven to have antimicrobial activity and xanthones are the main chemical constituents of this plant [22,23], the antibacterial activities of all isolated compounds (**1**–**11**) against *C. violaceum* ATCC12472 were screened. As a result (Table 2), compounds **7**, **9**, **10** and **11** manifested antibacterial activity with MIC ranging from 31.25 to 500 μM. Notably, all the tested compounds did not inhibit the growth of *C. violaceum* at subinhibitory concentrations of MIC. Based on the results of this study, it appeared that the antibacterial activity of simple xanthones was stronger than that of xanthonolignoids. The presence of the phenylpropane moiety may decrease the antibacterial activity. In simple xanthones, 6-OH is essential for the maintenance of antimicrobial effects.

**Figure 5:**
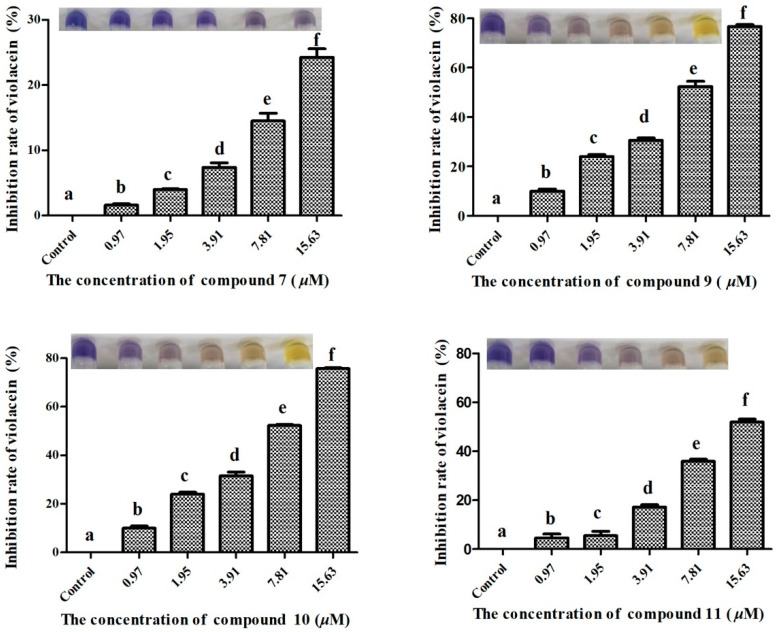
Inhibitory effects of compounds **7**, **9**, **10** and **11** on purple pigment formation of *C. violaceum* ATCC 12472. Note: The images shown are presented as the means ± standard deviation from three independent experiments in triplicates. The same letter indicates that the difference is not significant, and the different letters indicate significant differences.

Purple pigment formation is one of the main forms of cell-cell communication used by *C. violaceum* to coordinate their group behavior in response to population density. Compounds **7**, **9**, **10** and **11** were further assessed for if they can inhibit the purple pigment formation of *C. violaceum*. As shown in Figure 5, at the concentration of 16.53 μM, these compounds could reduce the formation of purple pigment with an inhibition rate of 21.27%, 75.36%, 76.21% and 53.11%, respectively.

**Figure 6:**
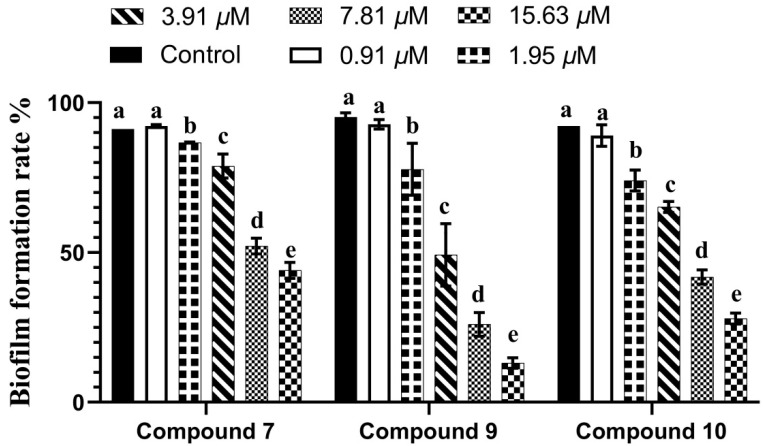
Inhibitory rate of compounds **7**, **9** and **10** on *C. violaceum* biofilm formation. Note: Biofilm formation of *C. violaceum* was quantified at OD 490 nm in presence of compounds at 0–15.63 µM 30 °C after 24 h in 96-well plates. Columns represent means ± standard deviations. Bars with different letters (a–e) differ significantly (*p* < 0.05).

Recent reports suggest that the formation of rigid biofilms on natural and artificial surfaces drastically promotes the resistance capabilities of bacteria. The combination of antibiotics with bacterial biofilm-preventing or disturbing agents seems to be a promising strategy to prevent bacterial infection [24]. The formation of bacterial biofilms is regulated by the QS system. In this study, the biofilm inhibition potential of compounds **7**, **9** and **10** was evaluated at different concentrations using crystal violet staining. As shown in Figure 6, the formation of biofilms increased with decreasing drug concentration in a classic dose-dependent manner. At a concentration of 15.63 µM, the biofilm inhibition rates of compounds **7**, **9** and **10** were 55.17%, 86.23% and 74.25%, respectively. These results suggest that compounds **7**, **9** and **10** have potential abilities to inhibit the formation of biofilms, which is considered a potential drug target for controlling drug-resistant chronic infections [25].

**Figure 7:**
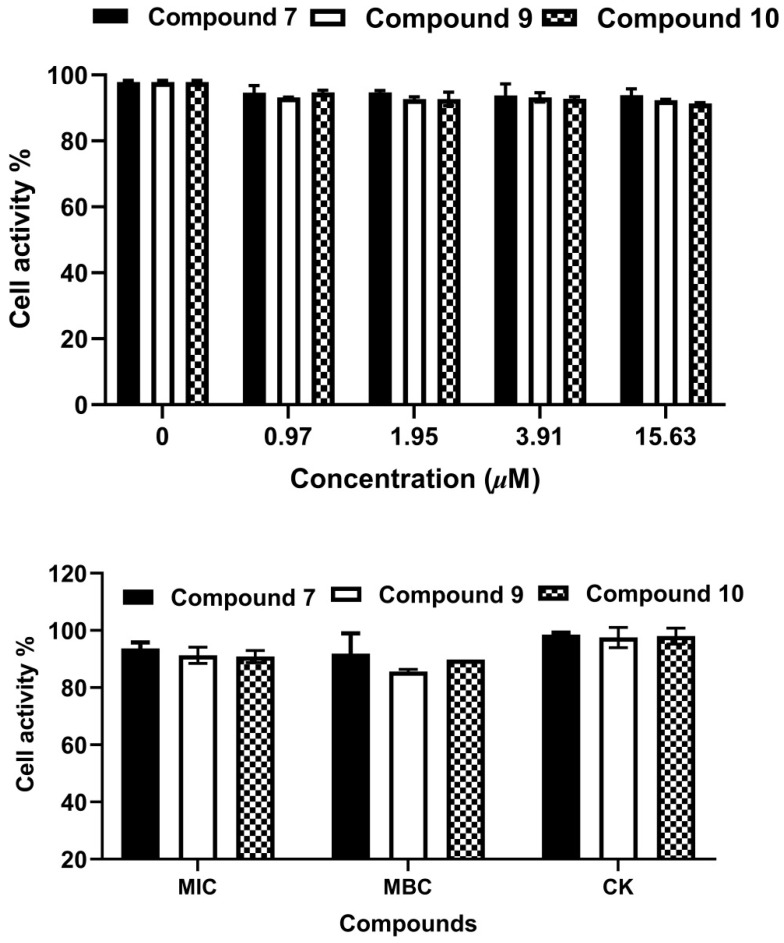
Cytotoxicity of compounds **7**, **9** and **10** on PC-12.

In light of several studies revealing the cytotoxic activity of xanthones, the cytotoxic effects of compounds **7**, **9** and **10** toward PC-12 cells were evaluated using an MTT assay. As summarized in Figure 7**,** the three compounds exhibited no cytotoxic activity on PC-12 cells at the concentration of 0.97–15.63 µM. It’s worth noting that compounds **7**, **9** and **10** showed more than 50% bacterial biofilm reduction at the concentration of 15.63 µM but were still safe for PC-12 cells. They showed no obvious damage to PC-12 cells even at the concentrations of MIC and MBC.

QS is a cell-density-dependent communication process to measure population density and trigger appropriate responses and conduct behavioral regulation [26]. QS inhibitors inhibit the QS system by reducing its virulence factor production and biofilm formation without affecting bacterial growth, thus making it difficult to cause drug resistance [27]. The above results signified that bacterial killing was not responsible for the reduction of biofilm formation, and compounds **7**, **9** and **10** acted as quorum sensing inhibitors rather than antimicrobial agents. These compounds inhibit QS of *C. violaceum* at sub-inhibitory concentrations representing their unique mechanism for anti-quorum sensing other than growth inhibition or cell death, which was congruent with previous findings [9,28]. Purple pigment and biofilm inhibition potential demonstrated that they act by disturbing the bacterial communication system and attenuating microbial pathogenicity without killing the pathogens. Owing to the insufficient amounts, further anti-quorum sensing mechanism of these compounds was not investigated.

## 4. Conclusions

As part of our ongoing investigation on natural QSIs, two new xanthonolignoids (**1** and **2**) each existing as a racemic mixture, one new simple oxygenated xanthone (**7**) and eight known analogs (**3**–**6**, **8**–**11**) were obtained from *H. scabrum*. Chiral separation of **1** yielded a pair of enantiomers **1a** and **1b**. The structures of these compounds were elucidated by spectroscopic analysis and ECD calculations. The evaluation for the QS inhibitory activity against *C. violaceum* indicated that both **9** and **10** exhibited the most potent QS inhibitory effects with the minimum inhibitory concentration (MIC) and minimum bactericidal concentration (MBC) values of 31.25 and 62.5 μM, respectively. The antibacterial activity of simple xanthones was stronger than that of xanthonolignoids. The presence of the phenylpropane moiety may decrease its antibacterial activity, and 6-OH in simple xanthones is essential for the maintenance of antimicrobial effect, which can be deduced from the analysis of structure–activity relationship. Crystal violet staining was employed to determine the biofilm inhibition potential of compounds **7**, **9** and **10**. The results indicated that the formation of biofilms increased with decreasing drug concentration in a classic dose-dependent manner. The biofilm inhibition rates of these three compounds (15.63 µM) were 55.17%, 86.23% and 74.25%, respectively. The cytotoxic assay results suggested that the three compounds exhibited no cytotoxic activity on PC-12 cells at the concentration of 0.97–15.63 µM, MIC and MBC, which suggested that compounds **7**, **9** and **10** acted as quorum sensing inhibitors rather than antimicrobial agents. They act by inhibiting the formation of purple pigment and biofilm so as to disturb bacterial communication systems and attenuate microbial pathogenicity without killing the pathogens. These findings highlight the importance of the genus *Hepricum* as a source of QS inhibitory compounds.

## Figures and Tables

**Table 1:** ^1^H (500 MHz) and ^13^C NMR (125 MHz) data of compounds **1, 2, 7** and **8** (in DMSO-*d*_6_).

	1		2		7		8	
Position	*δ*_H_ (Multiplicity, *J* in Hz)	*δ*_C_	*δ*_H_ (Multiplicity, *J* in Hz)	*δ*_C_	*δ*_H_ (Multiplicity, *J* in Hz)	*δ*_C_	*δ*_H_ (Multiplicity, *J* in Hz)	*δ*_C_
1	8.00 d (8.5)	127.6	7.97 d (8.5)	127.5		147.4		152.3
2	6.86 dd (8.5, 1.5)	114.7	6.84 dd (8.5, 1.5)	114.9		148.2	6.51 d (8.0)	107.8
3		164.9		165.5	7.18 d (8.0)	122.7	7.12 d (8.0)	121.5
4	6.82 ^a^	101.9	6.82 ^a^	101.9	6.83 d (8.0)	105.4		137.0
4a		157.4		157.5		139.4		143.7
5		132.4		132.4		157.9		158.1
6		138.8		138.7	7.32 s	106.1	7.30 s	105.9
7		145.5		145.4		152.6		152.3
8	7.16 s	96.6	7.16 s	96.6	6.71 s	101.6	6.74 s	101.6
8a		113.9		114.0		109.8		110.1
9		173.7		173.6		180.0		179.5
9a		113.0		112.9		108.0		108.1
10a		141.0		140.9		144.7		145.1
1′		126.7		125.7				
2′	7.05 d (1.5)	112.1	6.77 s	105.7				
3′		147.7		148.0				
4′		147.3		136.2				
5′	6.82 ^a^	115.4		148.0				
6′	6.90 dd (8.0, 1.5)	120.8	6.77 s	105.7				
7′	5.05 d (7.5)	76.3	5.04 d (7.5)	76.6				
8′	4.37 m	77.8	4.42 m	77.7				
9′	3.70 dd (12.5, 1.5) 3.42 dd (12.5, 4.0)	59.9	3.72 d (12.5) 3.42 d (12.5)	59.9				
7-OCH_3_	3.84 s	55.8	3.85 s	55.7				
3′-OCH_3_	3.78 s	55.7	3.78 s	56.1				
5′-OCH_3_			3.78 s	56.1				

^a^ overlapped signals.

**Table 2:** The MIC and MBC values of 11 compounds from *Hypericum scabrum* against *C. violaceum* ATCC 12472.

Compounds	MIC (μM)	MBC (μM)	MBC/MIC Ratio
**1a**	>1000	>1000	–
**1b**	>1000	>1000	–
**2**	>1000	>1000	–
**3**	>1000	>1000	–
**4**	>1000	>1000	–
**5**	>1000	>1000	–
**6**	>1000	>1000	–
**7**	250	500	2
**8**	>1000	>1000	–
**9**	31.25	62.5	2
**10**	31.25	62.5	2
**11**	125	250	2
kanamycin	13.73	27.46	2

## Data Availability

Not applicable.

## Supplementary Materials

The following supporting information can be downloaded at https://www.mdpi.com/article/10.3390/molecules27175519/s1, Figure S1: 1H NMR (500 MHz, DMSO-*d*_6_) spectrum of compound **1**; Figure S2: 13C NMR (125 MHz, DMSO-*d*_6_) spectrum of compound **1**; Figure S3: HRESIMS spectrum of compound **1**; Figure S4: HSQC spectrum of compound **1**; Figure S5: HMBC spectrum of compound **1**; Figure S6: 1H NMR (500 MHz, DMSO-*d*_6_) spectrum of compound **2**; Figure S7: ^13^C NMR (125 MHz, DMSO-*d*_6_) spectrum of compound **2**; Figure S8: HRESIMS spectrum of compound **2**; Figure S9: HSQC spectrum of compound **2**; Figure S10: HMBC spectrum of compound **2**; Figure S11: ^1^H NMR (500 MHz, DMSO-*d*_6_) spectrum of compound **7**; Figure S12: ^13^C NMR (125 MHz, DMSO-*d*_6_) spectrum of compound **7**; Figure S13: HRESIMS spectrum of compound **7**; Figure S14: HSQC spectrum of compound **7**; Figure S15: HMBC spectrum of compound **7**; Figure S16: ^1^H NMR (500 MHz, DMSO-*d*_6_) spectrum of compound **8**; Figure S17: 13C NMR (125 MHz, DMSO-*d*_6_) spectrum of compound **8**; Figure S18: HRESIMS spectrum of compound **8**; Figure S19: HSQC spectrum of compound **8**; Figure S20: HMBC spectrum of compound **8.**

## Sample Availability

Samples of the compounds are not available from the authors.
