# Supplementary material for: Three New Xanthones from Hypericum scabrum and Their Quorum Sensing Inhibitory Activities against Chromobacterium violaceum
Source: Molecules. 2022 Aug 27;27(17):5519. doi: 10.3390/molecules27175519 (PMC9458047; doi:10.3390/molecules27175519)

Supplementary Material

Three new xanthones from *Hypericum scabrum* and their quorum sensing inhibitory activities against *Chromobacterium violaceum*

Li-Ping Teng[1,2,†], Hong Zeng[1,†], Cai-Yan Yang[2], He-Bin Wang[3,*] and Zhong-Bo Zhou[1,2,*]

[1] Research Center for the Prevention and Treatment of Drug Resistant Microbial Infecting, Youjiang Medical University for Nationalities, Baise 533000, PR China

[2] School of Pharmacy, Youjiang Medical University for Nationalities, 98 Chengxiang Road, Baise 533000, PR China

[3] College of Chemical Engineering and Technology, Tianshui Normal University, Tianshui, 741000, PR China

* Correspondence: zzb7855@163.com (Z.-B.Z.); wanghebin@tsnu.edu.cn (H.-B.W.)

† These authors contributed equally to this work.

List of Figures

Figure S1 ^{1}H NMR (500 MHz, DMSO-d_6) spectrum of compound **1**

Figure S2 ^{13}C NMR (125 MHz, DMSO-d_6) spectrum of compound **1**

Figure S3 HRESIMS spectrum of compound **1** **Figure S4** HSQC spectrum of compound **1**

Figure S5 HMBC spectrum of compound **1**

Figure S6 ^{1}H NMR (500 MHz, DMSO-d_6) spectrum of compound **2**

Figure S7 ^{13}C NMR (125 MHz, DMSO-d_6) spectrum of compound **2**

Figure S8 HRESIMS spectrum of compound **2** **Figure S9** HSQC spectrum of compound **2**

Figure S10 HMBC spectrum of compound **2**

Figure S11 ^{1}H NMR (500 MHz, DMSO-d_6) spectrum of compound **7**

Figure S12 ^{13}C NMR (125 MHz, DMSO-d_6) spectrum of compound **7**

Figure S13 HRESIMS spectrum of compound **7**

Figure S14 HSQC spectrum of compound **7**

Figure S15 HMBC spectrum of compound **7**

Figure S16 ^{1}H NMR (500 MHz, DMSO-d_6) spectrum of compound **8**

Figure S17 ^{13}C NMR (125 MHz, DMSO-d_6) spectrum of compound **8**

Figure S18 HRESIMS spectrum of compound **8**

Figure S19 HSQC spectrum of compound **8**

Figure S20 HMBC spectrum of compound **8**

Figure S1 ^{1}H NMR (500 MHz, DMSO-d_6) spectrum of compound **1**

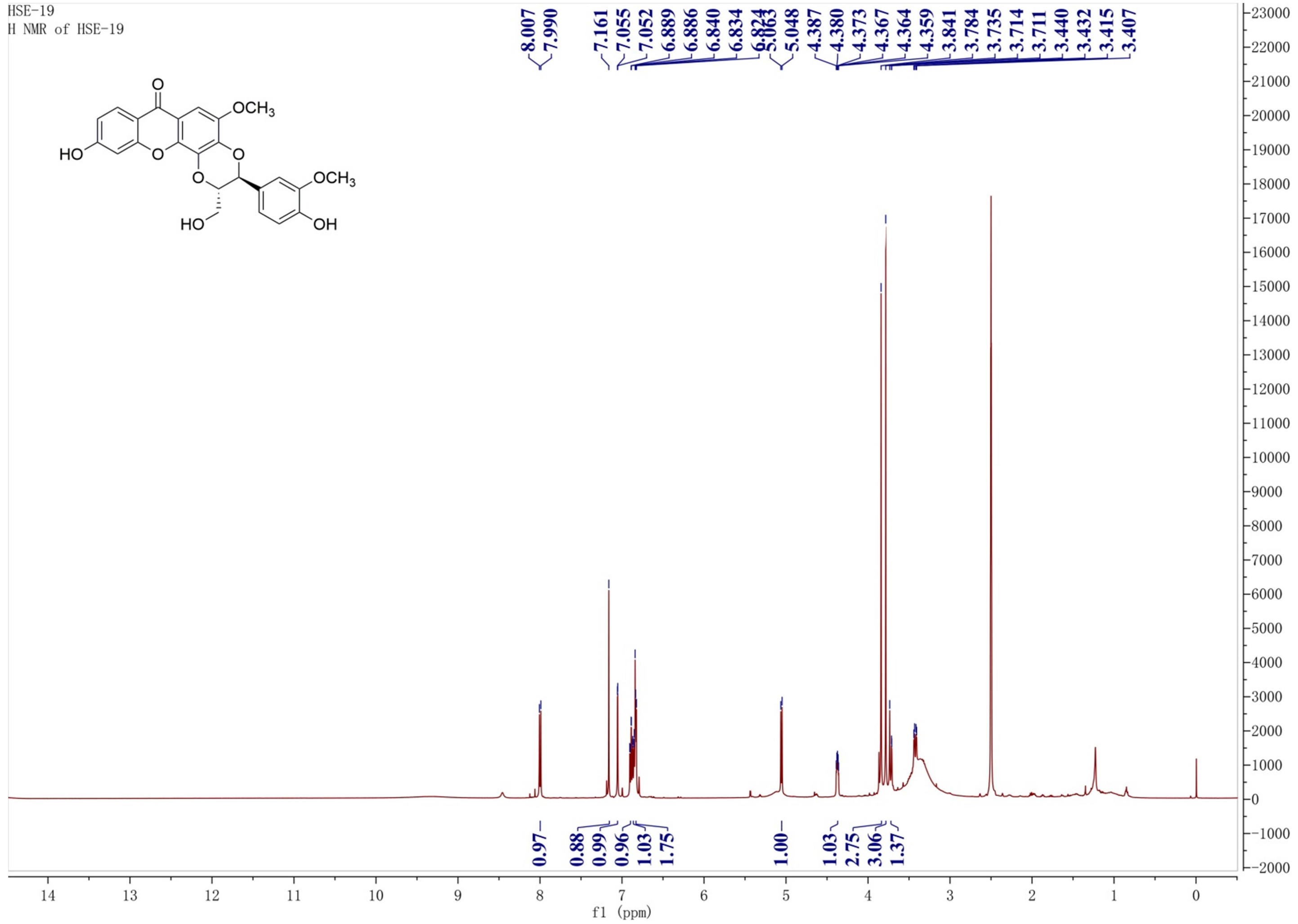

Figure S2 ^{13}C NMR (125 MHz, DMSO-d_6) spectrum of compound **1**

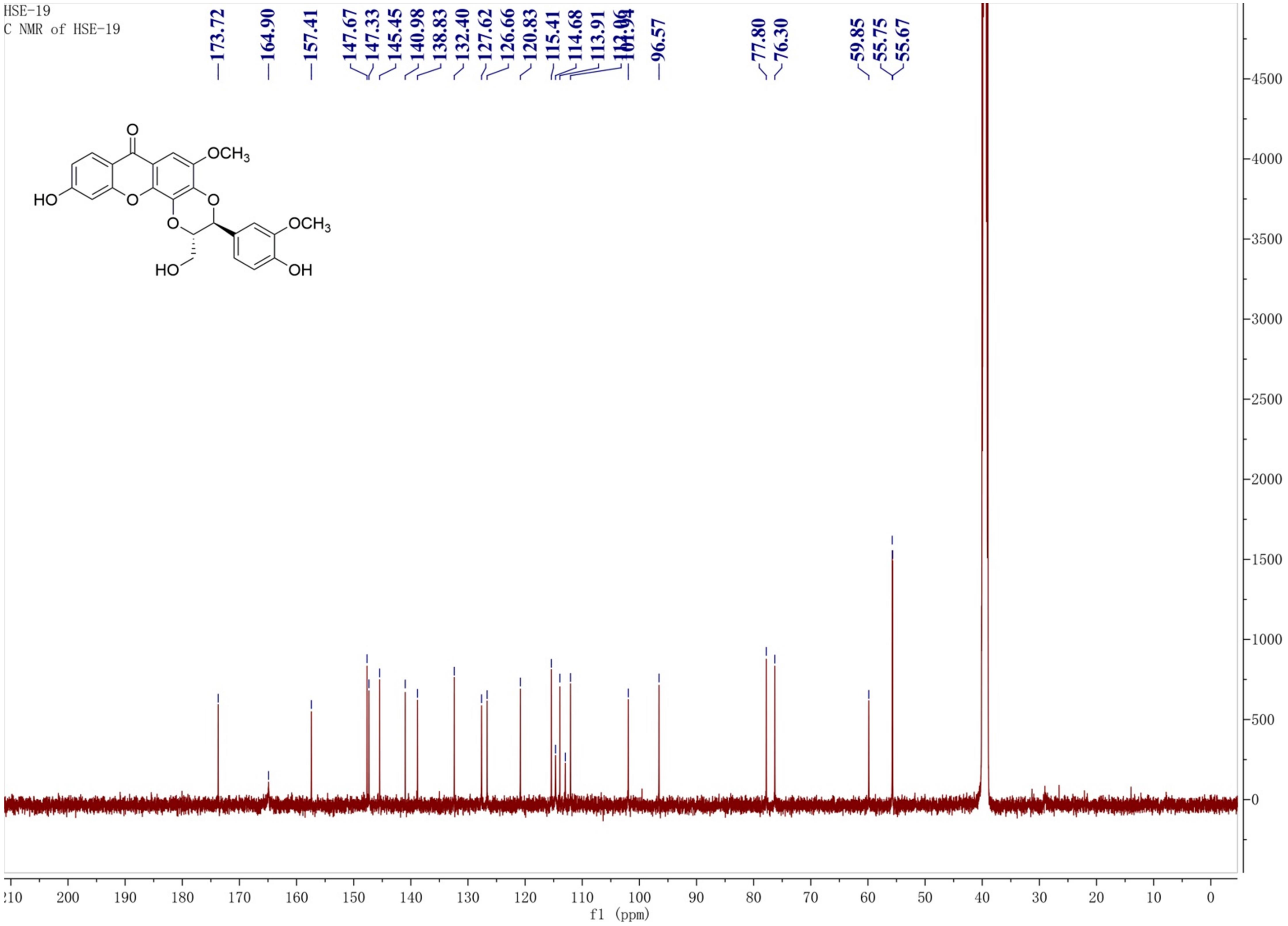

Figure S3 HRESIMS spectrum of compound **1**

**Analysis Name:**	**HSE-19-1**	**Acquisition Date:**	**20201230**
**Comment:**	**ESI Positive**	**Operator:**	**TIPC**

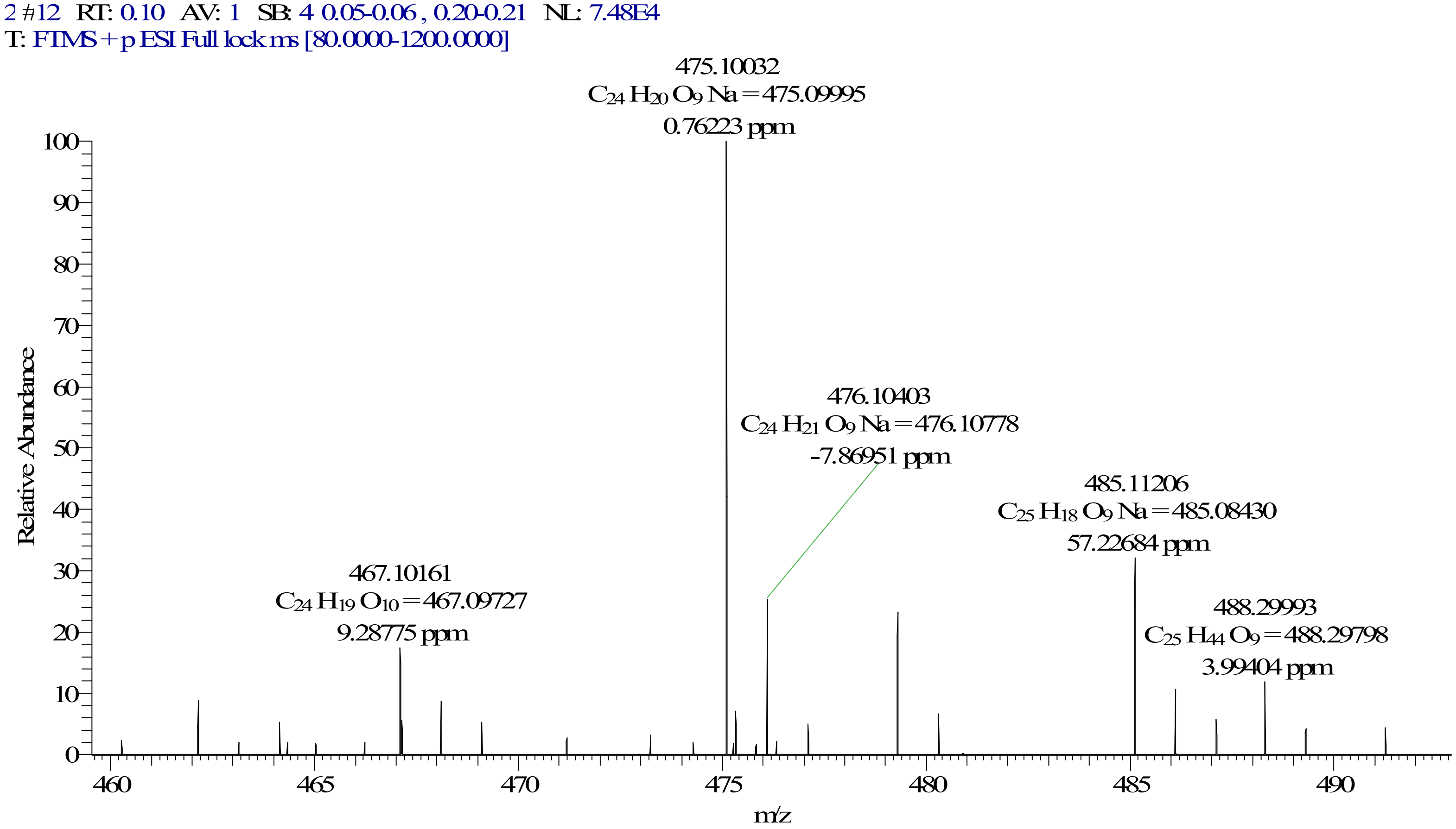

Figure S4 HSQC spectrum of compound **1**

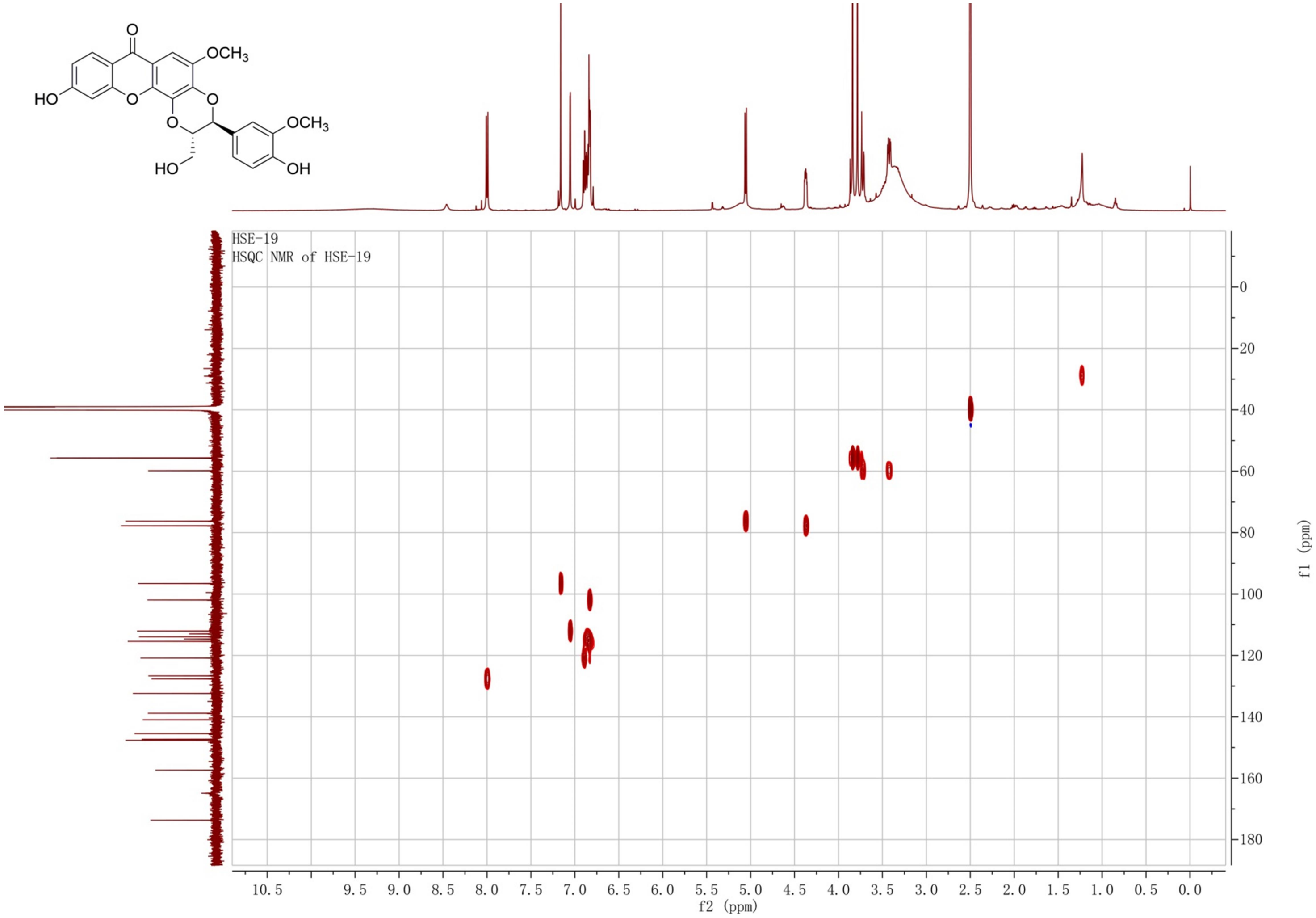

Figure S5 HMBC spectrum of compound **1**

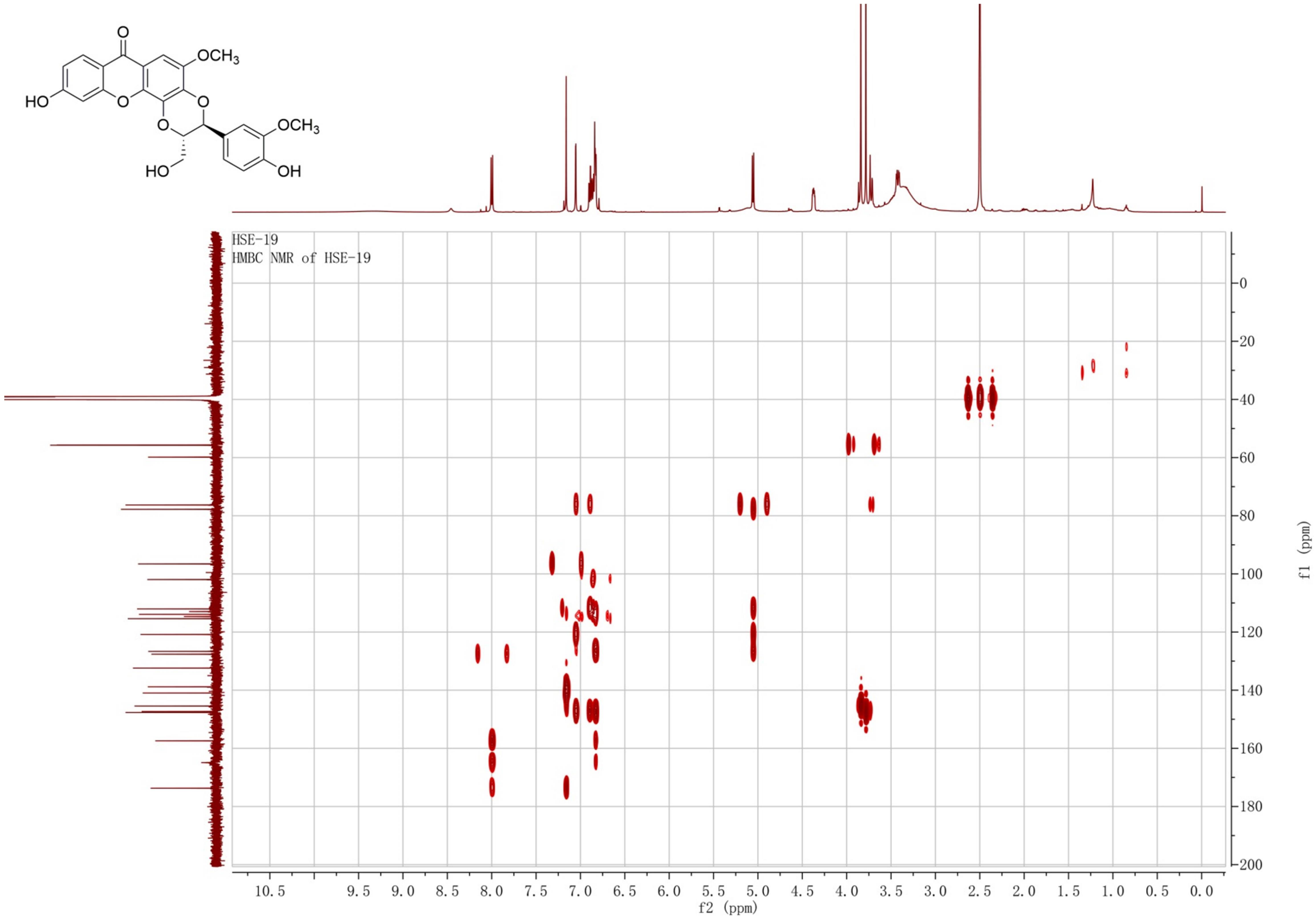

Figure S6 ^{1}H NMR (500 MHz, DMSO-d_6) spectrum of compound **2**

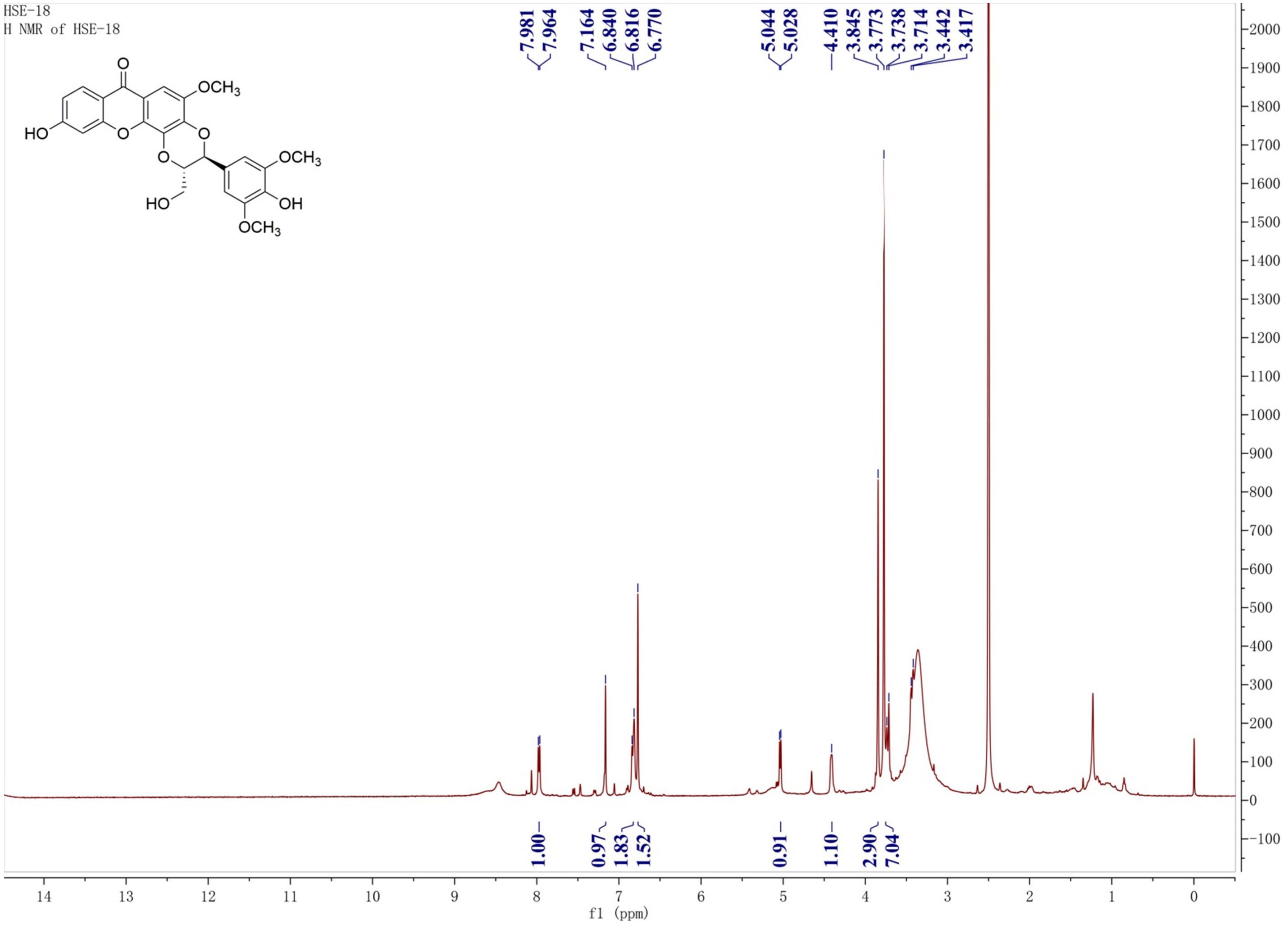

Figure S7 ^{13}C NMR (125 MHz, DMSO-d_6) spectrum of compound **2**

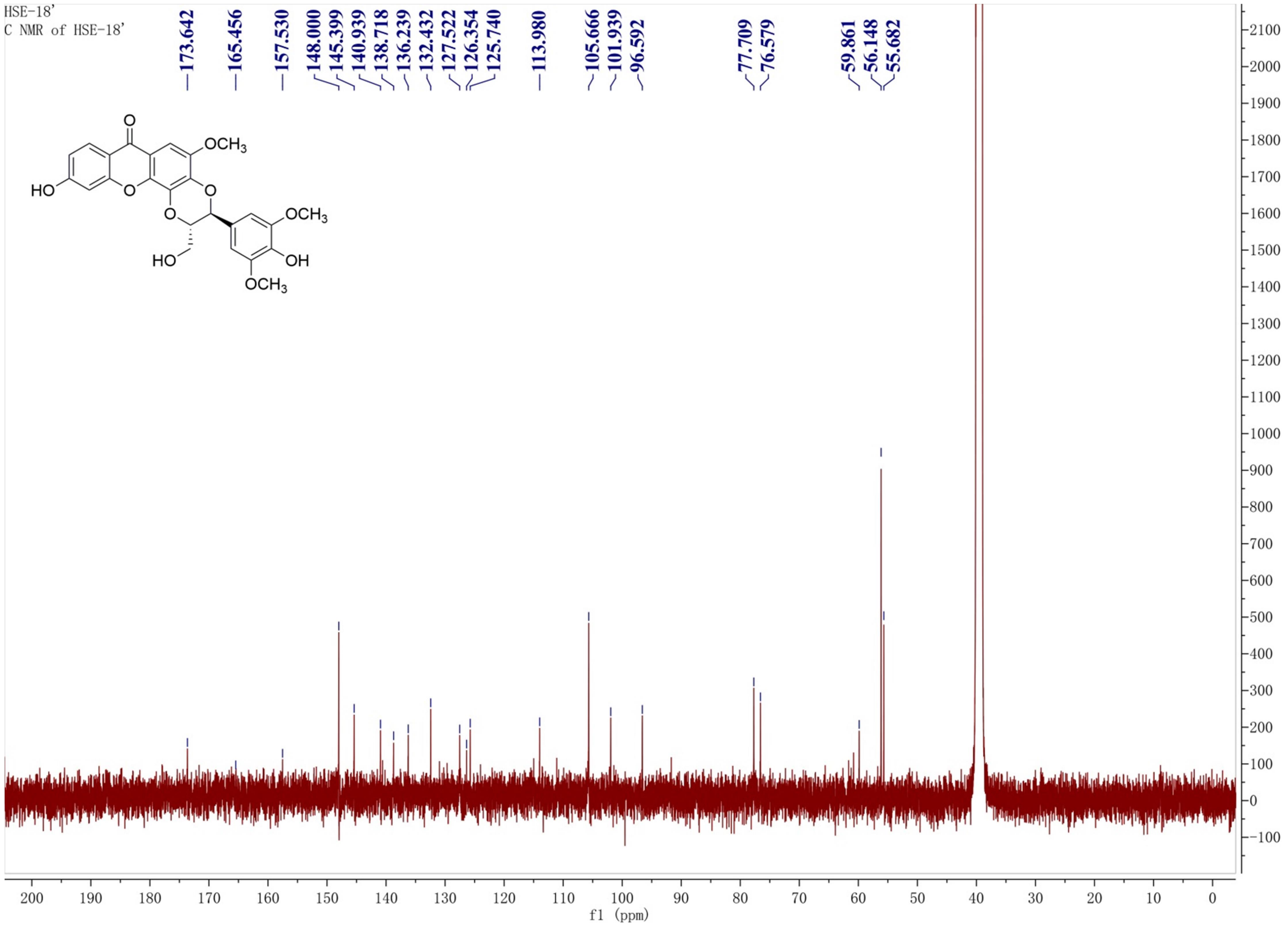

Figure S8 HRESIMS spectrum of compound **2**

**Analysis Name:**	**HSE-18**	**Acquisition Date:**	**20201230**
**Comment:**	**ESI Positive**	**Operator:**	**TIPC**

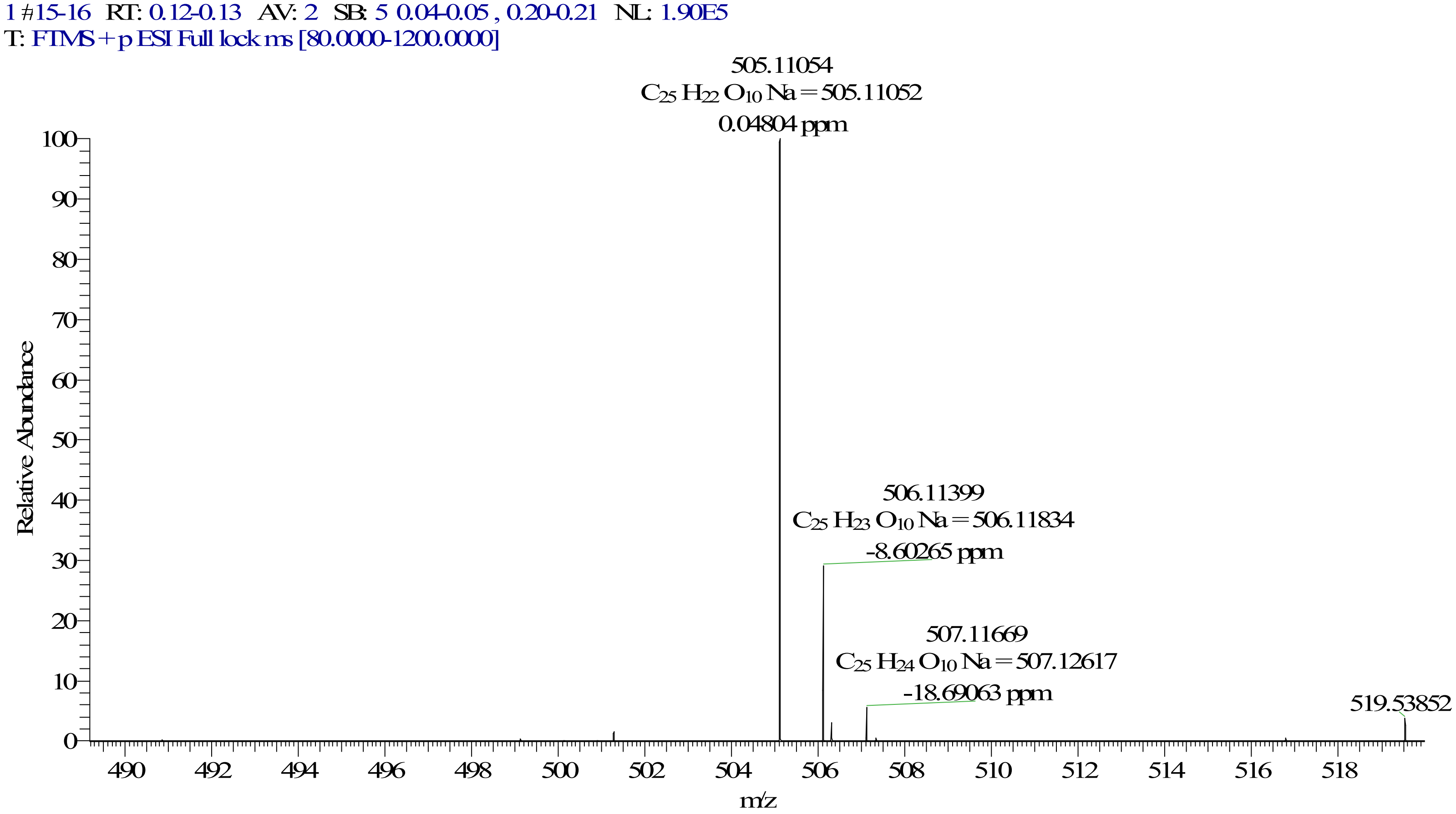

Figure S9 HSQC spectrum of compound **2**

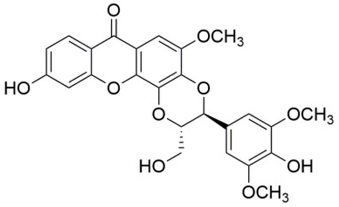

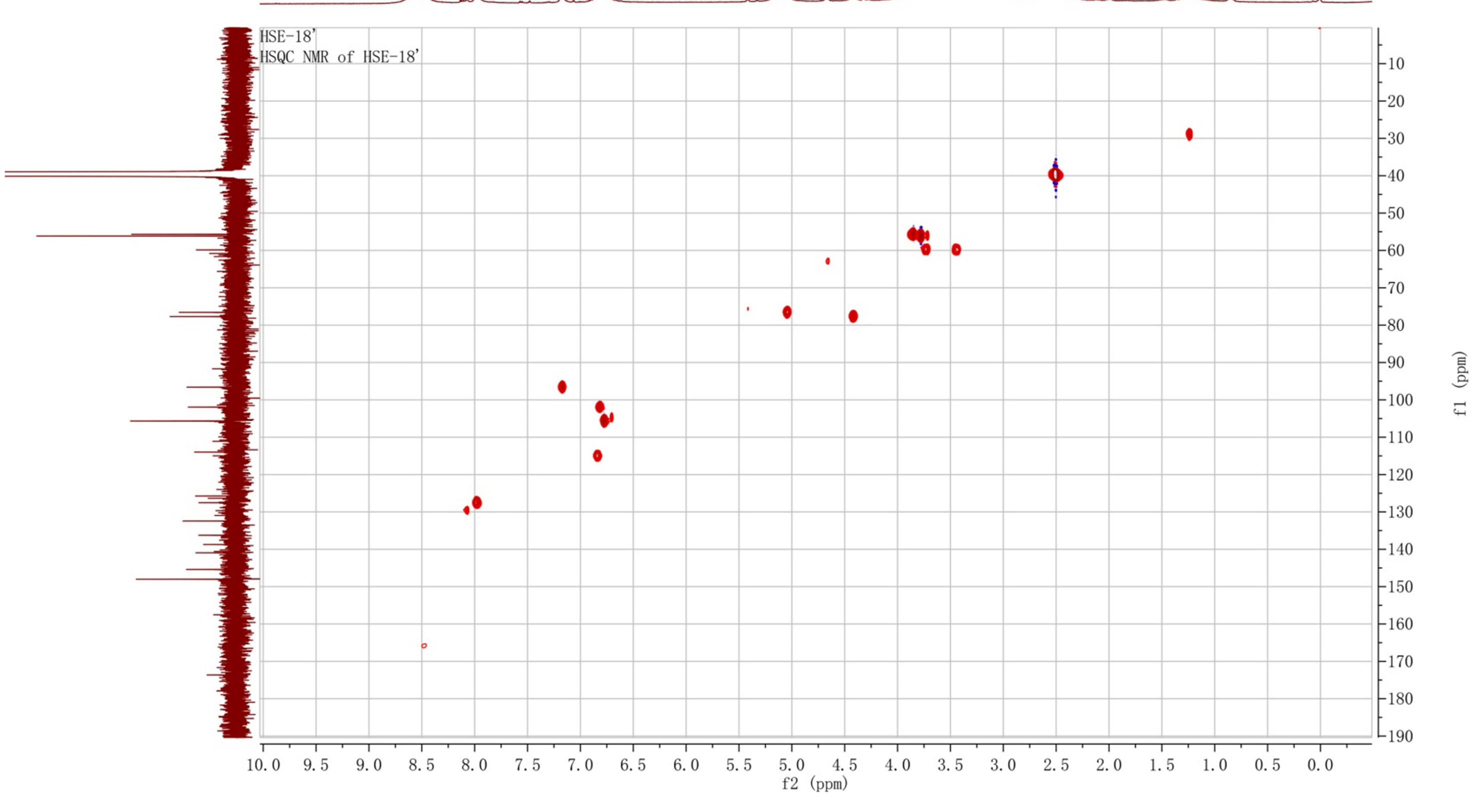

Figure S10 HMBC spectrum of compound **2**

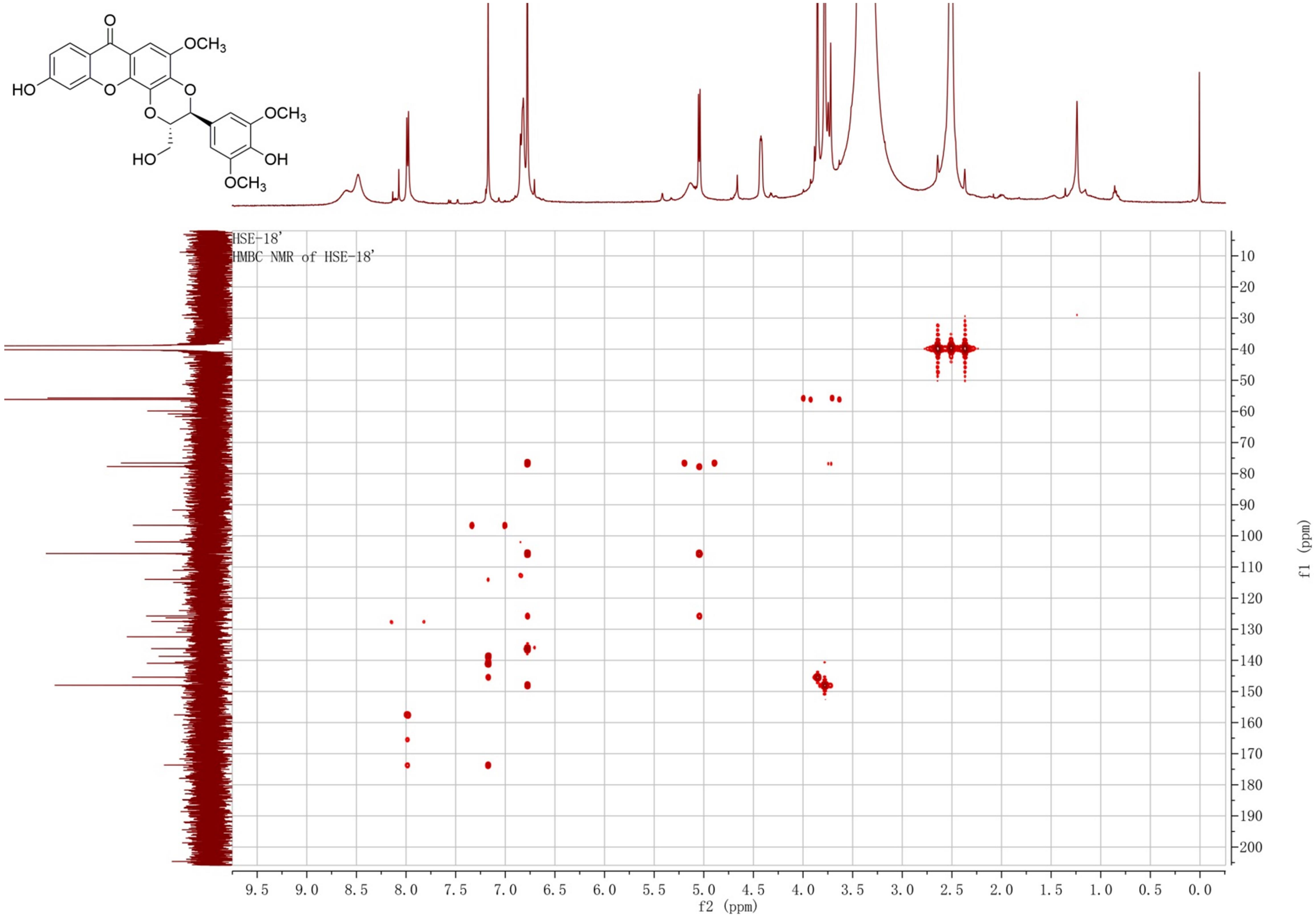

Figure S11 ^{1}H NMR (500 MHz, DMSO-d_6) spectrum of compound **7**

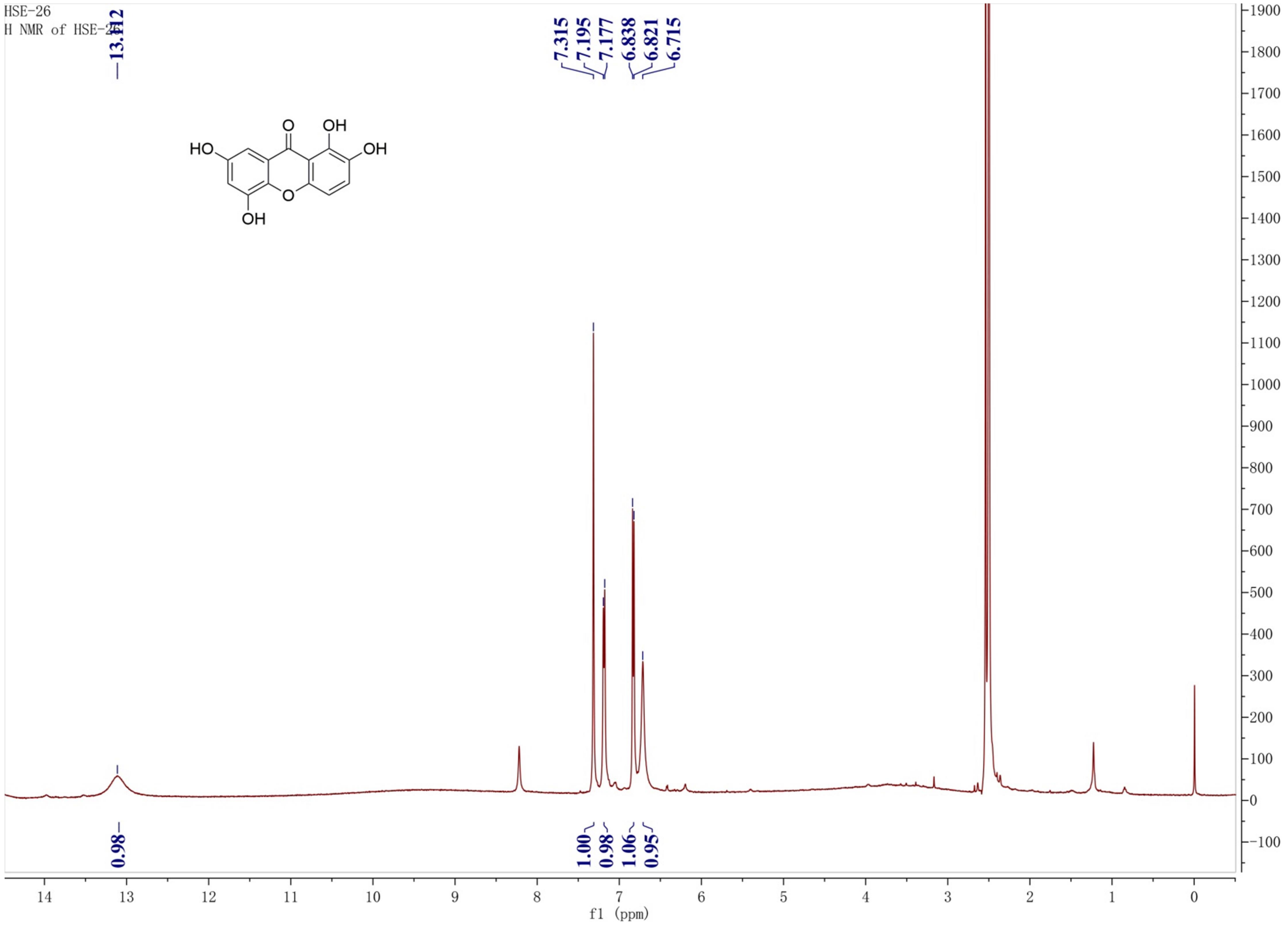

Figure S12 ^{13}C NMR (125 MHz, DMSO-d_6) spectrum of compound **7**

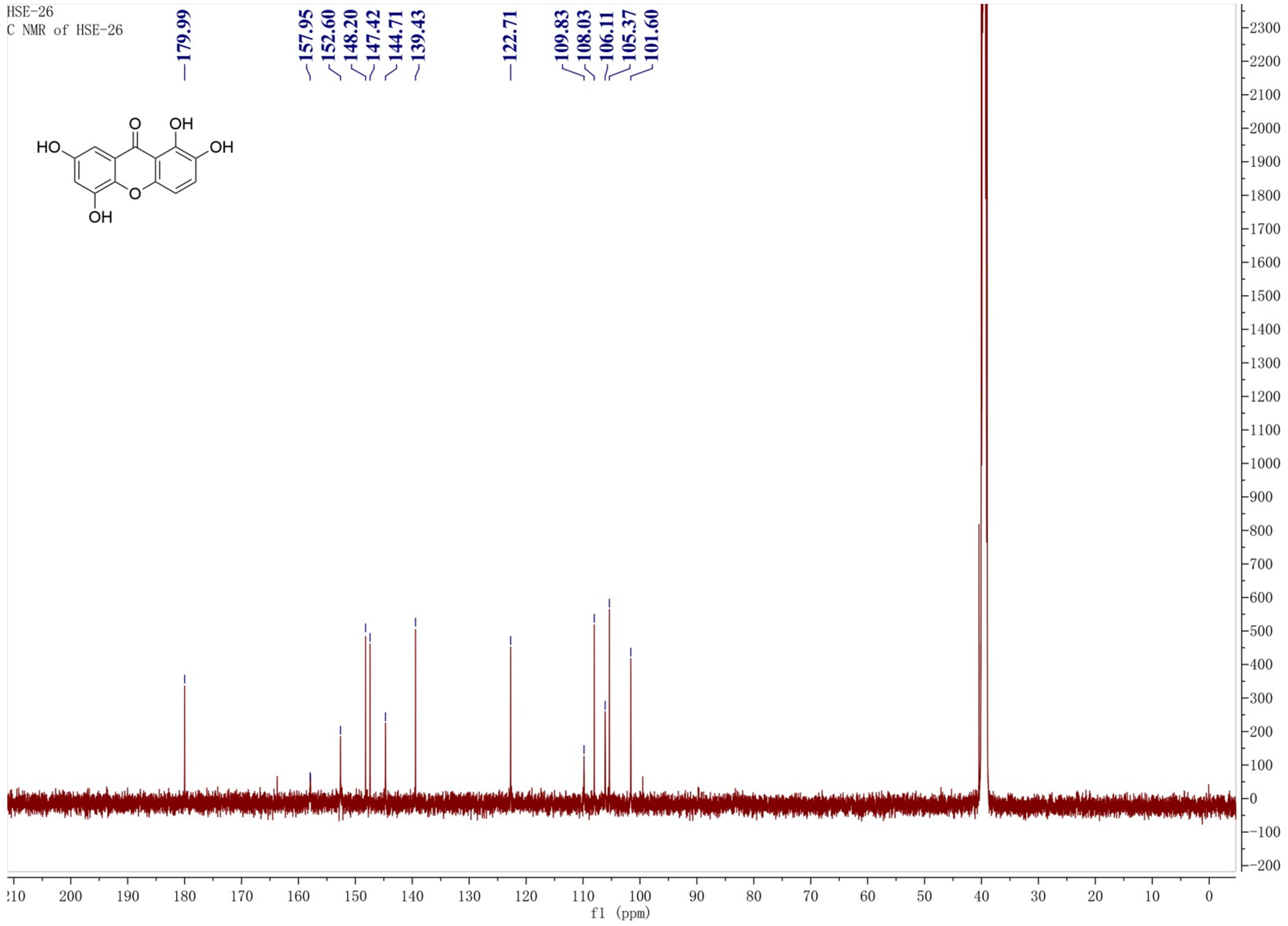

Figure S13 HRESIMS spectrum of compound **7**

**Analysis Name:**	**HSE-26**	**Acquisition Date:**	**20201230**
**Comment:**	**ESI Negative**	**Operator:**	**TIPC**

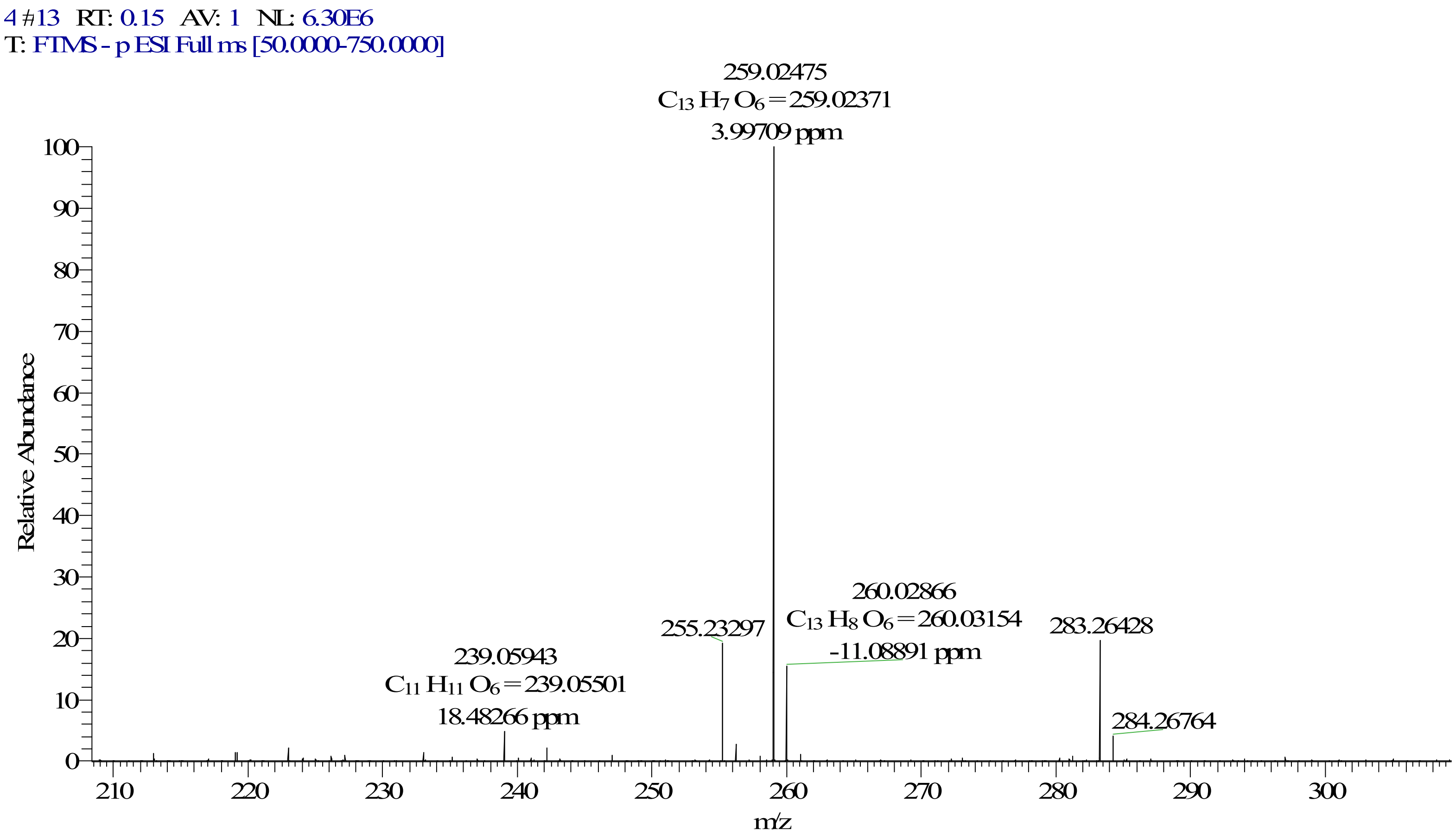

Figure S14 HSQC spectrum of compound **7**

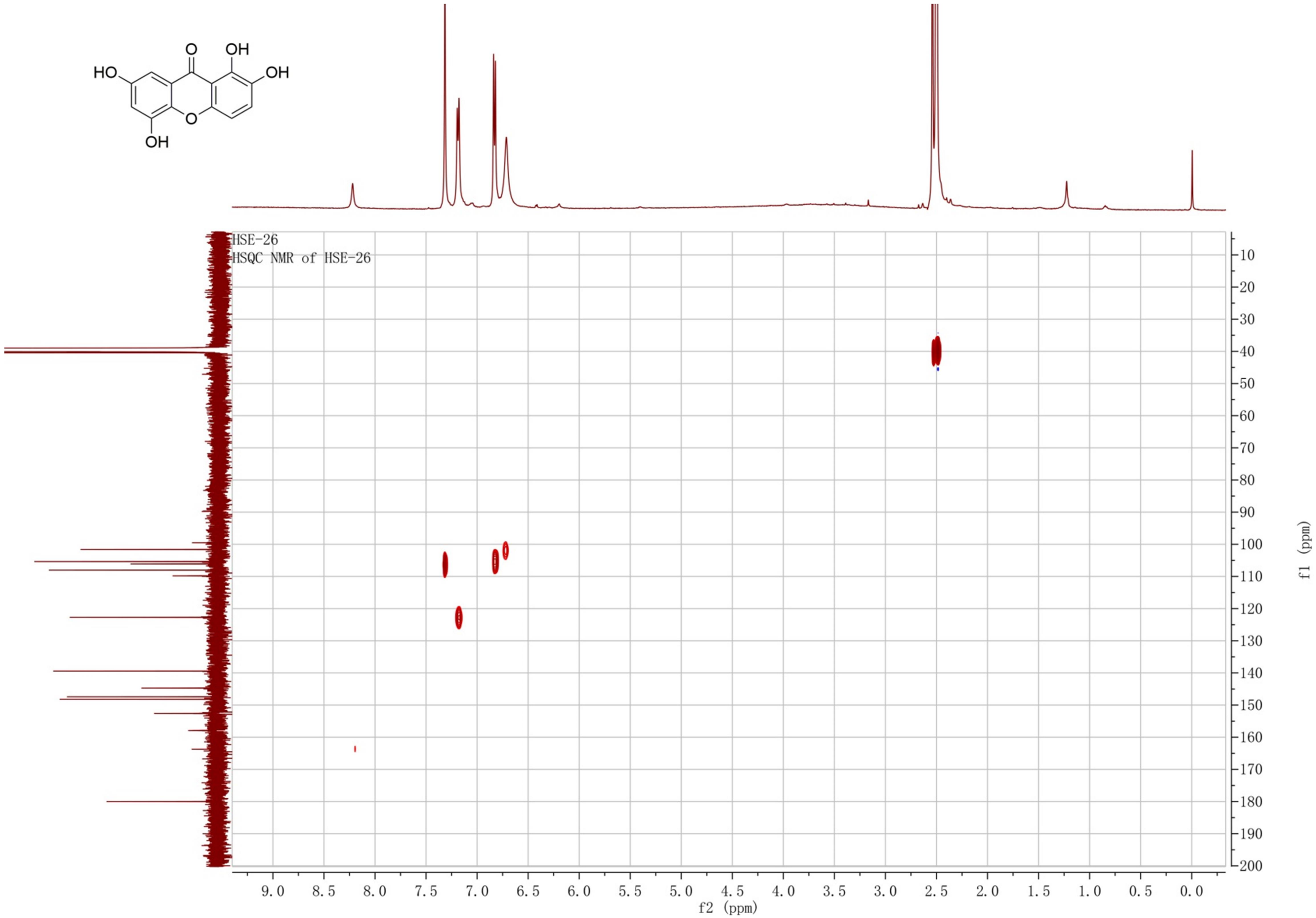

Figure S15 HMBC spectrum of compound **7**

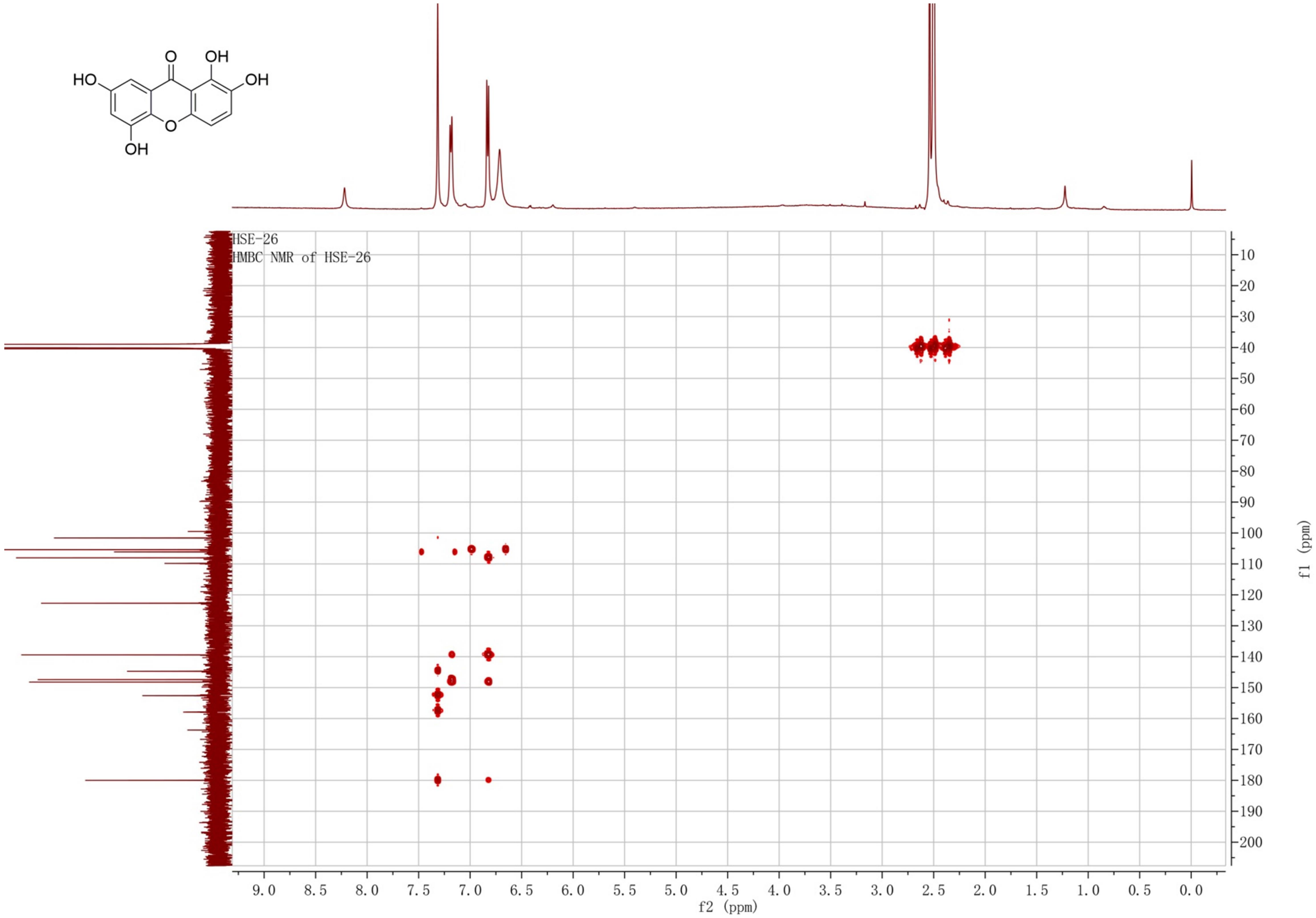

Figure S16 ^{1}H NMR (500 MHz, DMSO-d_6) spectrum of compound **8**

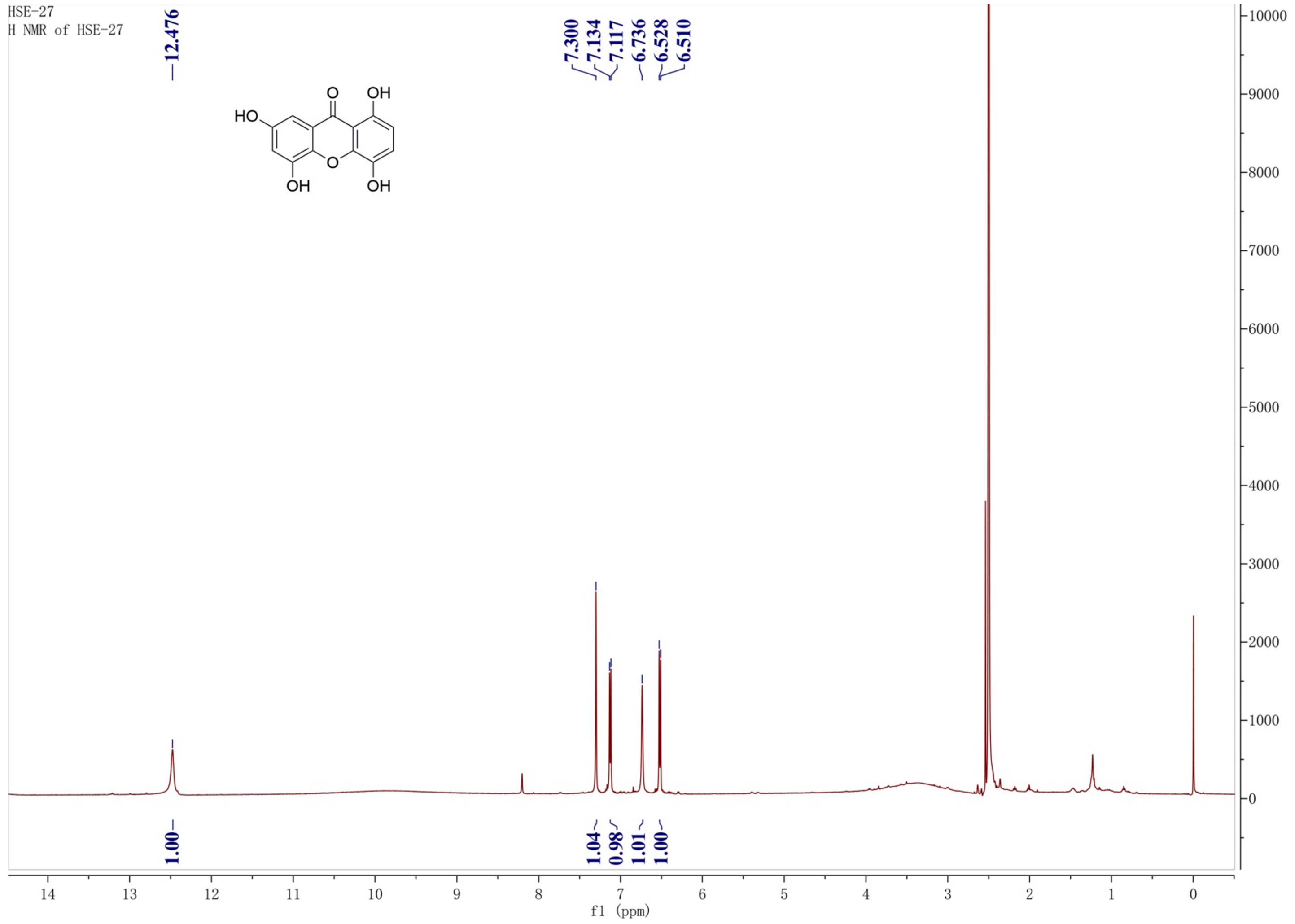

Figure S17 ^{13}C NMR (125 MHz, DMSO-d_6) spectrum of compound **8**

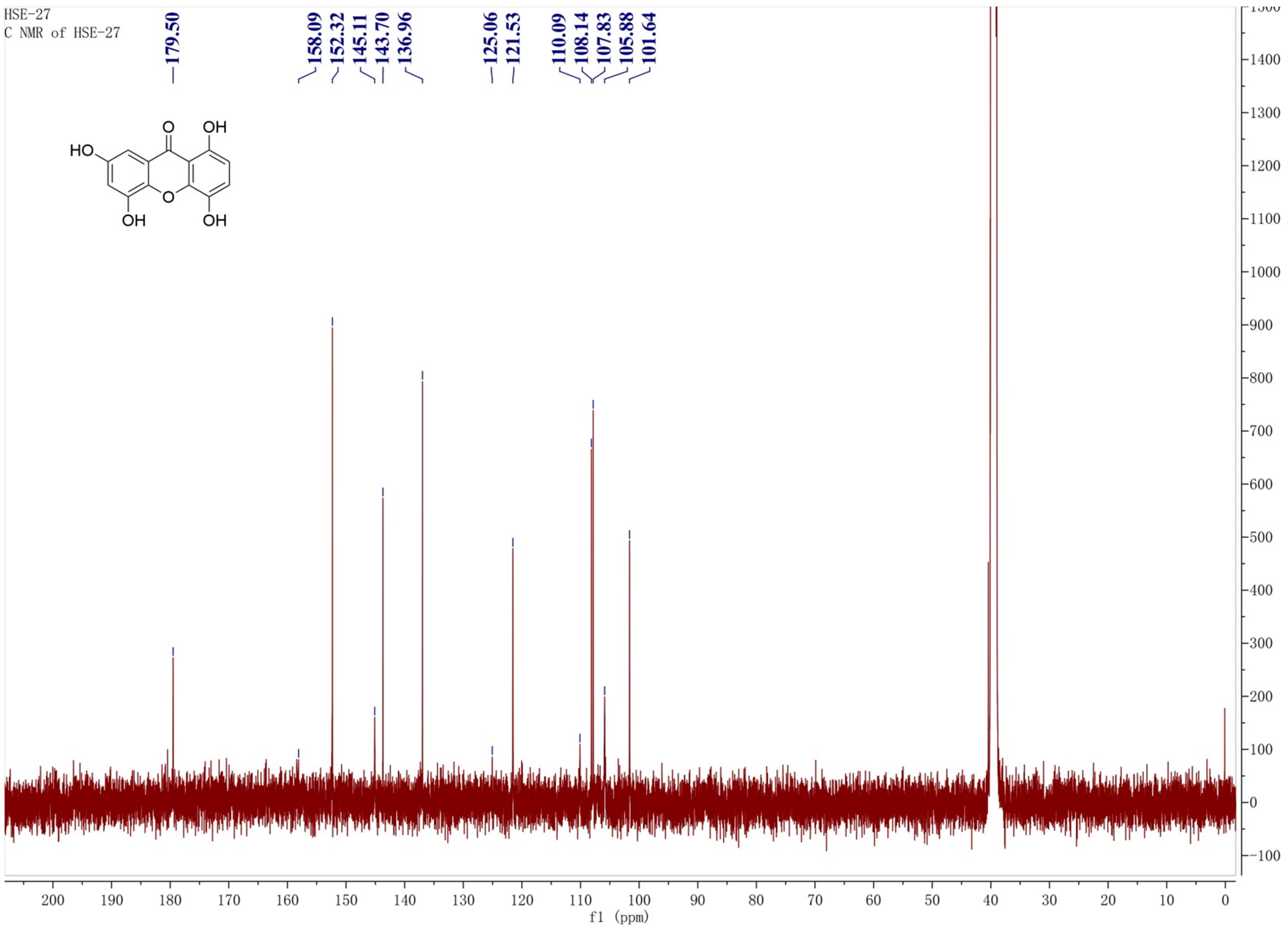

Figure S18 HRESIMS spectrum of compound **8**

**Analysis Name:**	**HSE-27**	**Acquisition Date:**	**20201230**
**Comment:**	**ESI Negative**	**Operator:**	**TIPC**

5 #11 RT: 0.13 AV: 1 SB: 1 0.05 , 0.22-0.23 NL: 3.76E6
T: FTMS - p ESI Full ms [50.0000-750.0000]

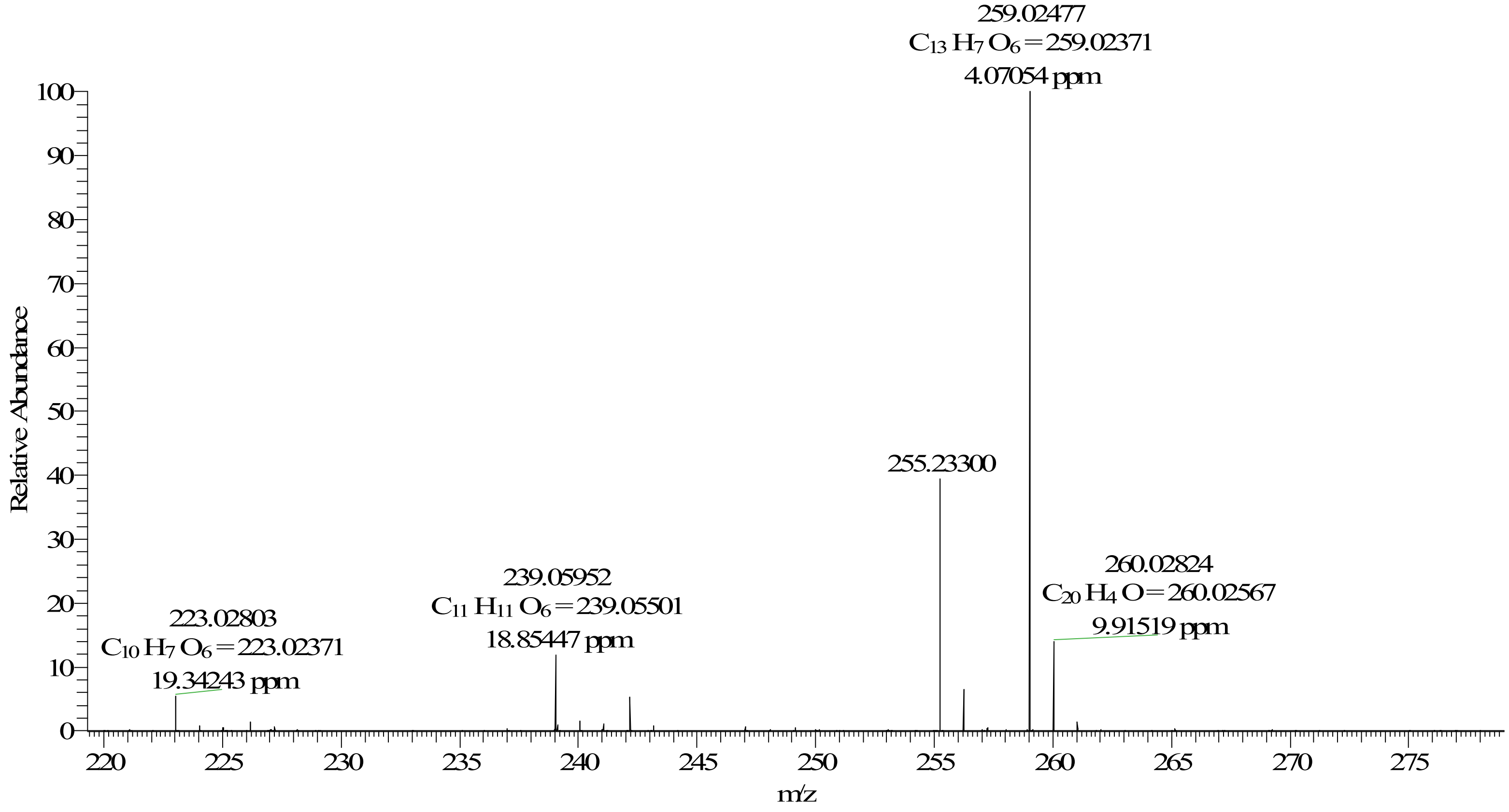

Figure S19 HSQC spectrum of compound **8**

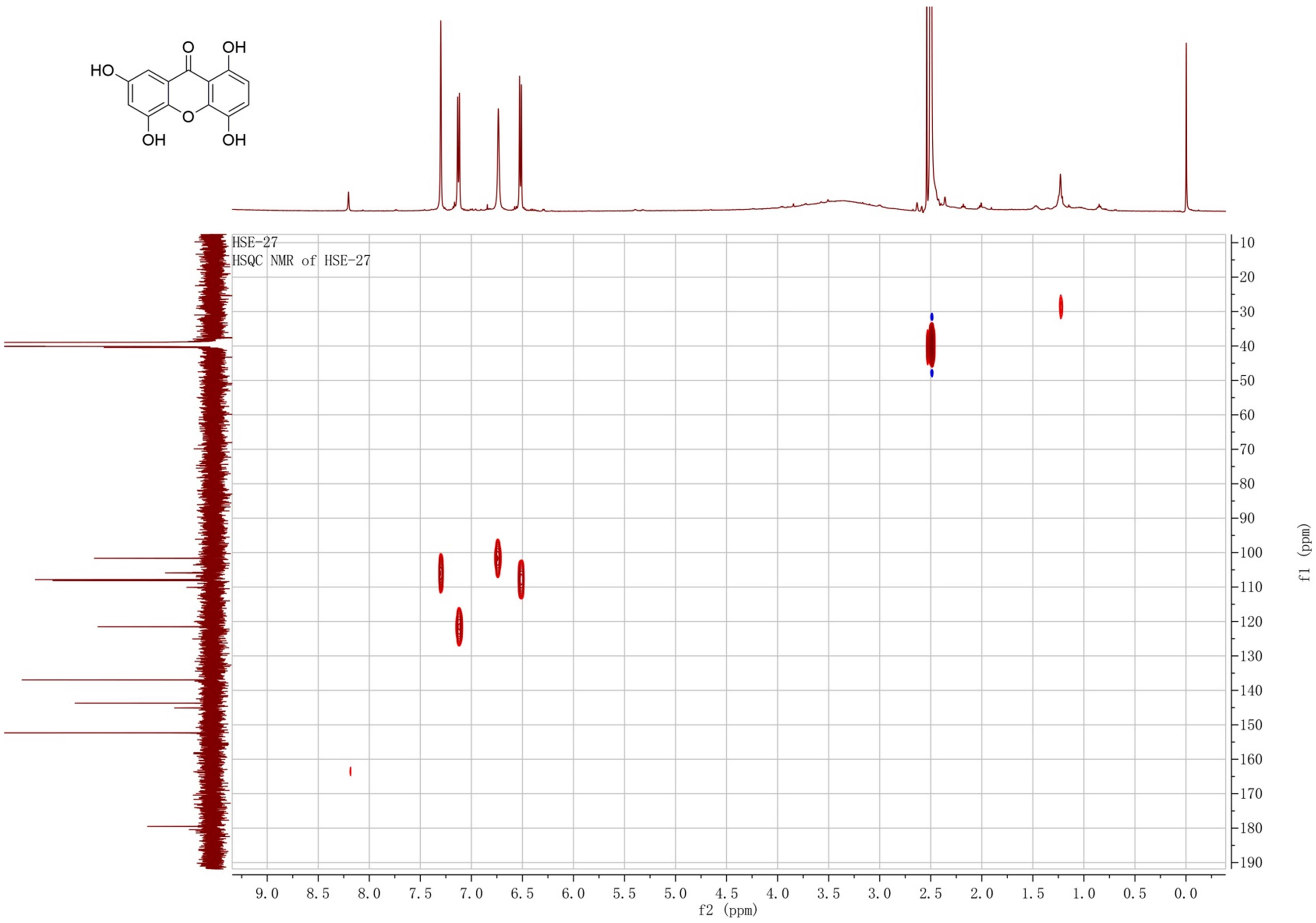

Figure S20 HMBC spectrum of compound **8**

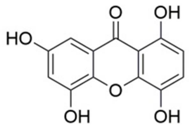

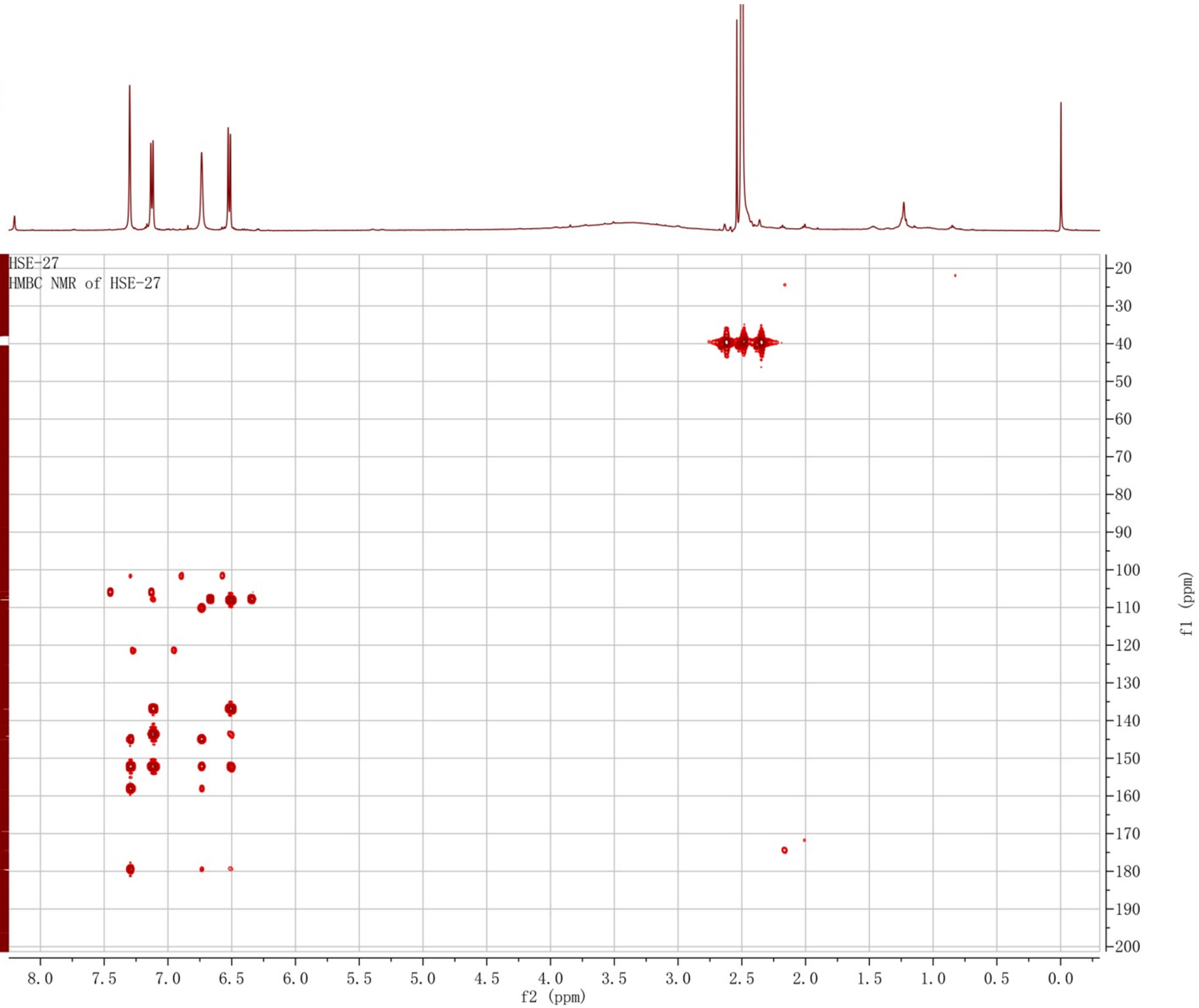

Supplement: Supplementary file 1 [file molecules-27-05519-s001.zip › molecules-1849479-supplementary.pdf]
